# Fructose-1,6-diphosphate inhibits viral replication by promoting the lysosomal degradation of HMGB1 and blocking the binding of HMGB1 to the viral genome

**DOI:** 10.1371/journal.ppat.1012782

**Published:** 2024-12-18

**Authors:** Penghui Hu, Huiyi Li, Zemin Ji, Weijia Jing, Zihan Li, Sujun Yu, Xiao Shan, Yan Cui, Baochen Wang, Hongyuan Dong, Yanzhao Zhou, Zhe Wang, Hui Xiong, Xiaomei Zhang, Hui-chieh Li, Jinrong Wang, Jiuzhou Tang, Ting Wang, Keliang Xie, Yuping Liu, Haizhen Zhu, Qiujing Yu

**Affiliations:** 1 Department of Critical Care Medicine, Tianjin Medical University General Hospital, Tianjin Institute of Immunology, State Key Laboratory of Experimental Hematology, Key Laboratory of Immune Microenvironment and Disease (Ministry of Education), Department of Immunology, School of Basic Medical Sciences, Tianjin Medical University, Tianjin, China; 2 Key Laboratory of Tropical Translational Medicine of Ministry of Education, Department of Pathogen Biology, School of Basic Medicine and Life Science, Department of Clinical Laboratory of the Second Affiliated Hospital of Hainan Medical University, The University of Hong Kong Joint Laboratory of Tropical Infectious Diseases, Hainan Medical University, Hainan, China; 3 Tianjin Institute of Immunology, State Key Laboratory of Experimental Hematology, Key Laboratory of Immune Microenvironment and Disease (Ministry of Education), Department of Immunology, School of Basic Medical Sciences, Tianjin Medical University, Tianjin, China; 4 Department of Health Management Centre & Institute of Health Management, Sichuan Provincial People’s Hospital, University of Electronic Science and Technology of China, Chengdu, China; 5 Department of Medical Oncology, The Affiliated Cancer Hospital of Zhengzhou University & Henan Cancer Hospital, Zhengzhou, China; 6 University of Electronic Science and Technology of China, Chengdu, China; 7 The Province and Ministry Co-sponsored Collaborative Innovation Center for Medical Epigenetics, School of Basic Medical Sciences, Department of Pharmacology and Tianjin Key Laboratory of Inflammation Biology, Tianjin Medical University, Tianjin, China; 8 Department of Critical Care Medicine, Tianjin Medical University General Hospital, Department of Anesthesiology, Tianjin Institute of Anesthesiology, Tianjin Medical University General Hospital, Tianjin, China; Cleveland Clinic Florida, UNITED STATES OF AMERICA

## Abstract

Fructose-1,6-diphosphate (FBP), a key glycolytic metabolite, is recognized for its cytoprotective effects during stress. However, the role of FBP in viral infections is unknown. Here, we demonstrate that virus-infected cells exhibit elevated FBP levels. Exogenous FBP inhibits both RNA and DNA virus infections *in vitro* and *in vivo*. Modulating intracellular FBP levels by regulating the expression of the metabolic enzymes FBP1 and PFK1 significantly impacts viral infections. Mechanistically, the inhibitory effects of FBP are not a result of altered viral adhesion or entry and are largely independent of type I interferon-mediated immune responses; rather, they occur through modulation of HMGB1. During viral infections, FBP predominantly reduces the protein levels of HMGB1 by facilitating its lysosomal degradation. Furthermore, FBP interacts with HMGB1 and disrupts the binding of HMGB1 to viral genomes, thereby further inhibiting viral replication. Our findings underscore the potential of FBP as a therapeutic target for controlling viral infections.

## Author summary

Viral infectious diseases pose a significant threat to public health, leading to widespread illness, hospitalizations, and fatalities. Developing effective treatments is crucial for alleviating the burden of these diseases on individuals and communities. In this study, we uncovered a novel role for fructose-1,6-diphosphate (FBP), a glycolytic metabolite, in suppressing viral replication. FBP achieves this by promoting the lysosomal degradation of HMGB1 and interacting with HMGB1 to prevent its binding to the viral genome. Due to its well-documented cytoprotective effects during periods of stress, FBP has been utilized as a supplemental treatment in various countries for heart and brain conditions associated with oxygen deprivation or compromised blood supply. Our findings suggest a promising new therapeutic strategy for treating viral infectious diseases through the repurposing of FBP.

## Introduction

Viral replication can be divided into five consecutive stages: adhesion, entry, uncoating, replication, and assembly and release [1, 2]. During the adhesion stage, the virus attaches to the cell surface, after which it enters the cytoplasm through fusion or pinocytosis. Once inside the cell, lysosomal enzymes in the phagosome degrade the viral capsid. Subsequently, the virus utilizes the host cell’s resources for nucleic acid replication and viral protein synthesis. Finally, complete viral particles are assembled and released, triggering further infections. Viral infections can cause varying degrees of damage or disease in the body, depending on the specific virus and the individual’s health status. The fatality rate of viral infectious diseases is a significant contributor to global mortality. Therefore, research on virus-host interactions is crucial for developing effective strategies to combat these diseases.

Host cells recognize viral RNA and DNA as damage-associated molecular patterns (DAMPs) through a variety of pattern recognition receptors (PRRs), including Toll-like receptors (TLRs), retinoic acid-inducible gene I (RIG-I)-like receptors, and cytosolic sensors of double-stranded DNA. Subsequently, PRRs recruit adaptor proteins such as TRIF, MAVS, and STING to activate the TBK1/IKKε kinases [3, 4]. TBK1/IKKε phosphorylates IRF3 and nuclear factor κB (NF-κB), facilitating their translocation to the nucleus and regulating the expression of type I interferon (IFN-I), such as IFN-α and IFN-β, as well as inflammatory cytokines [5, 6]. Once bound to the IFN-I receptor complex, IFN-I activates the JAK-STAT signaling pathway, which leads to the expression of interferon-stimulated genes (ISGs) that help combat viral infections and coordinate adaptive immunity [6].

During infection, viruses often exploit and reprogram cellular components to create an optimal environment for their replication. Viral infections can also modulate host cell metabolism, influencing viral survival or clearance [7–10]. Additionally, host cells may actively rewire their metabolic pathways to inhibit viral entry and replication, or to enhance antiviral immune responses [11–19]. For instance, when stimulated by RNA viruses, glycolytic metabolism is inhibited, leading to decreased lactate production. This reduction promotes the dissociation of lactate from the MAVS protein, facilitating the binding of RIG-I to MAVS. Consequently, this activates the RIG-I-MAVS signaling pathway, increasing IFN-I production and bolstering antiviral innate immunity [20].

Fructose-1,6-diphosphate (FBP) is a crucial metabolite in the glycolysis pathway. Fructose-6-phosphate kinase 1 (PFK1) catalyzes the conversion of fructose-6-phosphate (F6P) to FBP, then FBP undergoes a series of reactions to produce phosphoenolpyruvate (PEP) and pyruvate, which subsequently participate in glucose metabolism. The key enzyme in gluconeogenesis, fructose-1,6-bisphosphatase 1 (FBP1), catalyzes the decomposition of FBP into F6P and inorganic phosphate [21–23]. Research has demonstrated that FBP provides protective effects against cellular injury and plays a protective role in various tissues, including the brain, kidney, intestine, liver, and heart [24]. Due to its well-established cytoprotective properties during periods of stress, FBP has been employed in several countries as an adjunctive therapy for heart and brain conditions related to oxygen deprivation or reduced blood flow. Additionally, FBP is significant in immune responses. In a model of endotoxic shock induced by lipopolysaccharide (LPS), FBP inhibited the infiltration of inflammatory cells into lung tissue, reduced the expression of inflammatory factors, and exerted an anti-inflammatory effect [25]. However, it remains unclear whether FBP can regulate viral infections.

High-mobility-group box 1 (HMGB1) is a highly conserved and abundant non-histone chromosomal protein involved in various cellular processes, including DNA replication, transcription, recombination, and repair [26–29]. HMGB1 contains two homologous DNA/RNA binding domains, known as the A box and B box, each consisting of 75 amino acids, as well as a highly negatively charged C-terminal domain characterized by a sequence of extended glutamate and aspartic acid residues [30, 31]. As a DAMP, HMGB1 can trigger inflammatory responses by binding to PRRs such as TLRs [32]. Our recent study demonstrated that FBP reduces HMGB1 oligomerization by diminishing the interaction between the HMGB1 A-box and C-tail. This reduction in binding enhances the interaction between P53 and HMGB1 while decreasing HMGB1’s affinity for DNA. As a result, FBP exacerbates chemotherapy-induced cancer cell death [33]. Notably, FBP has been identified as a novel HMGB1 ligand and holds potential as an HMGB1-targeted cancer therapeutic [33].

In this study, we demonstrated that FBP can inhibit viral infection both *in vitro* and *in vivo*. This effect is not attributed to altered viral adhesion or entry and is largely independent of IFN-I signaling. HMGB1 plays a crucial role in the antiviral action of FBP; specifically, FBP reduces HMGB1 protein levels by promoting its lysosomal degradation. Furthermore, FBP directly interacts with HMGB1 and blocks the binding of HMGB1 to the viral genome, thereby further inhibiting viral replication. Overall, our findings highlight a novel approach for treating viral infectious diseases by repurposing FBP as a therapeutic agent.

## Results

### Exogenous FBP inhibits viral infection both *in vitro* and *in vivo*

**Fig 1:**
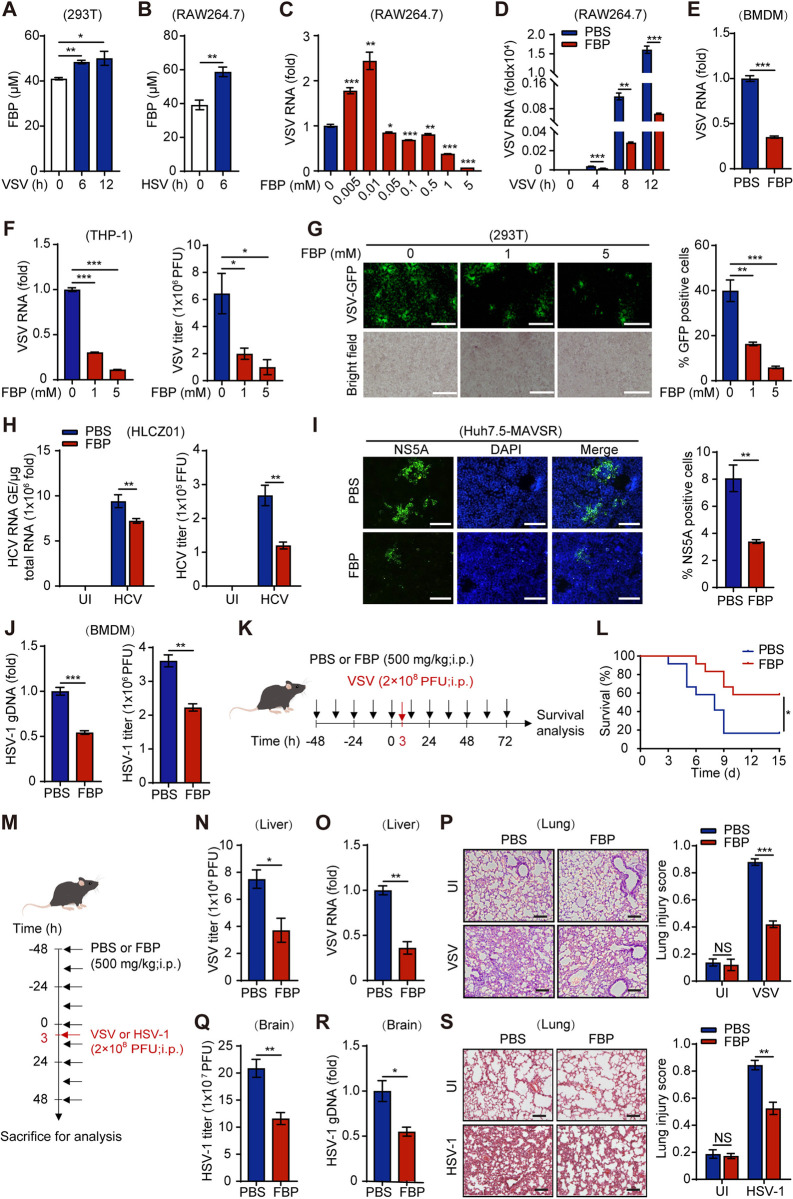
Exogenous FBP inhibits viral infection both *in vitro* and *in vivo*. (A and B) 293T cells or RAW264.7 cells were infected with VSV (MOI, 0.1) (A) or HSV-1 (MOI, 0.1) (B) for indicated times and the level of intracellular FBP was measured by colorimetric assay. (C and D) RAW264.7 cells were pretreated with the indicated concentrations of FBP (C) or 5 mM FBP (D) for 12 h and then infected with VSV (MOI, 0.1) for 10 h (C) or the indicated times (D). The cells were collected, and the RNA level of VSV was assessed by qPCR. (E and F) BMDMs (E) and THP-1 cells (F) were treated with the indicated concentrations of FBP for 12 h and infected with VSV (MOI, 0.1) for 10 h. VSV RNA was assessed by qPCR analysis and VSV titers were assessed by plaque assay. (G) 293T cells were treated with the indicated concentration of FBP for 12 h and infected with VSV-expressing green fluorescent protein (VSV-GFP) (MOI, 0.1) for 10 h. The cells were then observed under a fluorescence microscope (left panel; scale bars, 100 μm; right panel, quantification of the percentage of GFP-positive cells). (H) HLCZ01 cells were infected with HCV (MOI, 0.01) for 72 h and treated with 1 mM FBP for 12 h. HCV RNA was assessed by qPCR analysis and the HCV titers were determined as the average number of NS5A-positive foci. UI, Uninfected. (I) Huh7.5-MAVSR cells were infected with HCV (MOI, 0.01) for 72 h and treated with FBP (1 mM) for 12 h. Immunofluorescence using the indicated anti-NS5A antibody is shown (green). DAPI (blue) was used to counterstain the nuclei. (left panel, scale bars, 100 μm; right panel, quantification of the percentage of NS5A-positive cells). (J) BMDMs were treated with or without 1 mM FBP for 12 h and infected with HSV-1 (MOI, 0.1) for 10 h. HSV-1 gDNA was assessed by qPCR analysis and HSV-1 titers were assessed by plaque assay. (K) C57BL/6 mice were pretreated with FBP (500 mg/kg) or PBS (every 12 h for two days) and then intraperitoneally injected with VSV (2×10^8^ PFU per mouse). FBP or PBS was injected at the same frequency until the first mouse died during viral infection. (L) Survival of PBS and FBP mice at various times after intraperitoneal infection with VSV (2 ×10^8^ PFU per mouse) was monitored. (M) C57BL/6 mice were pretreated with FBP (500 mg/kg) or PBS (every 12 h for two days) and then intraperitoneally injected with VSV or HSV-1 (2×10^8^ PFU per mouse). The uninfected (UI) group was mock injected with PBS. FBP or PBS was injected at the same frequency during viral infection. (N-P) Mice were sacrificed at 24 h after infection for the analysis of VSV RNA via qPCR analysis (N) and the determination of the VSV titer in the liver via a TCID50 assay (O). Representative images of H&E-stained lung sections and quantification of the lung injury score are shown (scale bars, 100 μm) (P). (Q-S) Mice were sacrificed at 48 h after infection to assess HSV-1 gDNA by qPCR analysis (Q) and the HSV-1 titer, via a TCID50 assay, in the brain (R). Representative images of H&E-stained lung sections and quantification of the lung injury score are shown (scale bars, 100 μm) (S). Data are presented as the mean ± SEM. NS, not significant, *p < 0.05; **p < 0.01; ***p < 0.001, two-tailed Student’s t test. Differences in survival rates were analyzed using the log-rank (Mantel-Cox) test.

To investigate the effects of viral infections on FBP levels in cells, we measured FBP concentrations in the human embryonic kidney cell line 293T following infection with the RNA virus vesicular stomatitis virus (VSV), and in the mouse macrophage cell line RAW264.7 after infection with the DNA virus herpes simplex virus type 1 (HSV-1). Our results showed that viral infection significantly elevated intracellular FBP levels at 6–12 h (Fig 1A and 1B).

To investigate the role of FBP in viral infection, RAW264.7 macrophages and the human lung cell line A549 were pretreated with various concentrations of FBP before being infected with VSV (Figs 1C and S1A). We observed that exogenous FBP inhibited VSV RNA levels in a dose-dependent manner at higher concentrations (1–5 mM) (Figs 1C and 1D and S1A) and significantly increased intracellular FBP levels (S1B Fig).

Moreover, FBP not only inhibited VSV RNA levels and virus titers but also reduced the fluorescence of VSV-expressing green fluorescent protein (VSV-GFP) in various other mouse and human cell types, including primary mouse bone marrow-derived macrophages (BMDMs), the human monocytic cell line THP-1, the mammary tumor cell line E0771, and 293T cells (Figs 1E–1G and S1C).

Additionally, high doses of FBP (1–5 mM) conferred resistance to hepatitis C virus (HCV) infection in HLCZ01 human hepatoma cells, both in a JFH1 infection model and in Huh7.5-MAVSR cells that stably express a MAVS C508R mutant resistant to cleavage by the HCV-encoded NS3/4A serine protease (Figs 1H and S1D and S1E). Consistent with these findings, immunofluorescence analysis revealed a significant decrease in the fluorescence of NS5A, a nonstructural protein of HCV, in Huh7.5-MAVSR cells treated with FBP (Fig 1I). Additionally, FBP inhibited HSV-1 infection in various mouse and human cell lines, as evidenced by a reduction in HSV-1 genomic DNA (gDNA) and viral titers (Figs 1J and S1F–S1H).

We then conducted CCK-8 assays to investigate the effect of FBP on the viability of various cell types. Our results indicated that neither 1 mM nor 5 mM FBP exhibited cytotoxic effects during the 12–24 h treatment period (S1I–S1O Fig), suggesting that the inhibition of viral infection by FBP is attributed to its specific antiviral properties rather than nonspecific cytotoxicity.

To further elucidate the role of FBP in viral infections *in vivo*, we infected mice treated with either FBP or PBS with VSV or HSV. Mice receiving FBP exhibited lower mortality rates compared to those treated with PBS following VSV infection (Fig 1K and 1L). Additionally, FBP treatment resulted in a reduced VSV load in the liver and decreased HSV burden in the brain. FBP also diminished the infiltration of inflammatory cells and attenuated pathological changes in the lungs of virus-infected mice (Fig 1M–1S). Collectively, these findings demonstrate that FBP exerts a broad inhibitory effect on viral infections both *in vitro* and *in vivo*.

### FBP plays a crucial role in inhibiting viral infection

**Fig 2:**
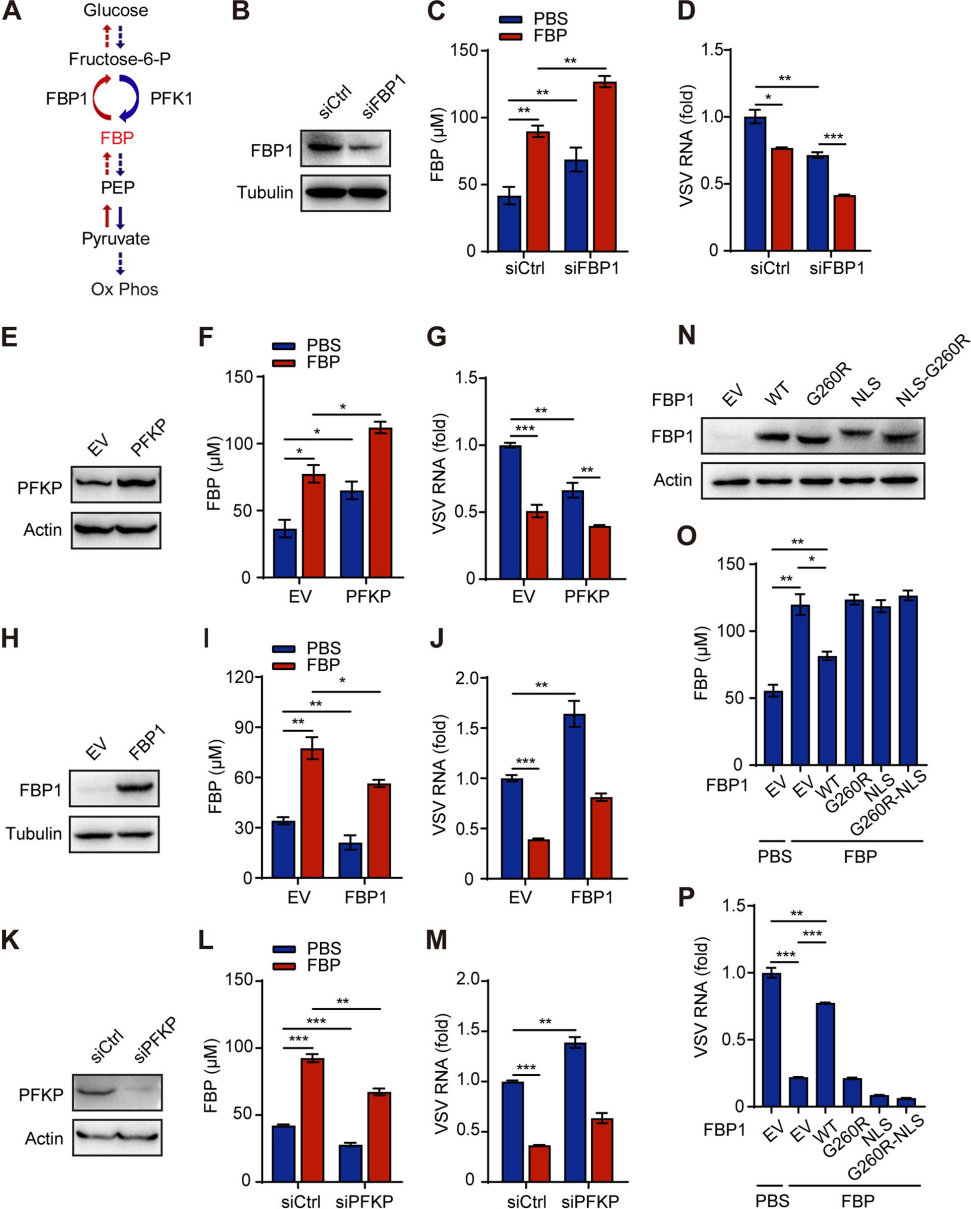
FBP plays a crucial role in inhibiting viral infection. (A) A simplified model describing the metabolic processes that regulate FBP production and consumption. FBP: Fructose-1,6-diphosphate, PFK1: Fructose-6-phosphate kinase 1, FBP1: Fructose-1,6-bisphosphatase 1, PEP: Phosphoenolpyruvate, Ox Phos: Oxidative Phosphorylation. (B-P) 293T cells were transfected with various constructs including siControl, siFBP1 (B-D), an empty vector (EV), PFKP-overexpressing plasmid (E-G), FBP1-overexpressing plasmid (H-J), siPFKP (K-M), FBP1 enzyme activity deletion mutant (FBP1-G260R), nuclear localization mutant (FBP1-NLS), and nuclear localization with activity deletion mutant (FBP1-NLS-G260R) (N-P). The cells were then treated with 5 mM FBP for 12 h and infected with VSV (MOI, 0.1) for 10 h. Western blot analysis was used to assess FBP1 and PFKP expression (B, E, H, K and N), intracellular FBP levels were measured using a microplate reader (C, F, I, L and O), and VSV RNA was assessed by qPCR (D, G, J, M and P). siCtrl, siControl. Data are presented as the mean ± SEM. NS, not significant, *p < 0.05; **p < 0.01; ***p < 0.001, one-way ANOVA.

To further elucidate the role of FBP in viral infection, we manipulated the expression of FBP1 and PFK1, which respectively regulate the consumption and production of FBP in cells (Fig 2A). Knocking down FBP1 resulted in a significant increase in intracellular FBP levels and inhibited VSV infection in 293T cells (Fig 2B–2D). The addition of exogenous FBP further elevated its intracellular concentration, enhancing this inhibitory effect (Fig 2B–2D). Similarly, overexpression of the PFK1 subunit PFKP yielded comparable results (Fig 2E–2G).

Conversely, overexpressing FBP1 significantly reduced intracellular FBP levels and enhanced VSV infection. The addition of exogenous FBP increased intracellular FBP levels and counteracted the increased infectivity (Fig 2H–2J). PFKP knockdown produced similar effects, reinforcing the connection between FBP levels and viral infection (Fig 2K–2M).

To further investigate the impact of FBP on viral infection, we generated various FBP1 mutants: an enzyme-deletion mutant (FBP1-G260R), a nuclear localization mutant (FBP1-NLS), and a combination of both features (FBP1-NLS-G260R) (Fig 2N). Wild-type FBP1 reduced intracellular FBP levels and facilitated VSV infection in 293T cells treated with exogenous FBP. However, the FBP1-overexpressing mutants did not produce similar results (Fig 2O and 2P), indicating that the enzymatic activity and non-nuclear localization of FBP1 is essential for its effect on VSV infection by altering FBP levels. Collectively, these data suggest that FBP itself plays a critical role in inhibiting viral infection, independent of the production of other glycolytic intermediates.

### FBP suppresses viral infection largely independent of the type I IFN signaling pathway

FBP can pass through the lipid bilayer of the cell membrane and affect its stability [34]. To investigate whether FBP inhibits VSV infection by interfering with viral adhesion and entry, we pretreated cells with FBP before infecting them with VSV at different temperatures for 1 hour: 4°C to assess the adhesion phase, 37°C for the entry phase, and a transition from 4°C to 37°C to evaluate both phases. Our results showed that FBP pretreatment enhanced the adhesion and entry of VSV and HSV but did not affect HCV (S2A–S2D Fig). These findings indicate that the inhibitory effect of FBP on viral infection is not mediated by a reduction in virus adhesion or entry.

**Fig 3:**
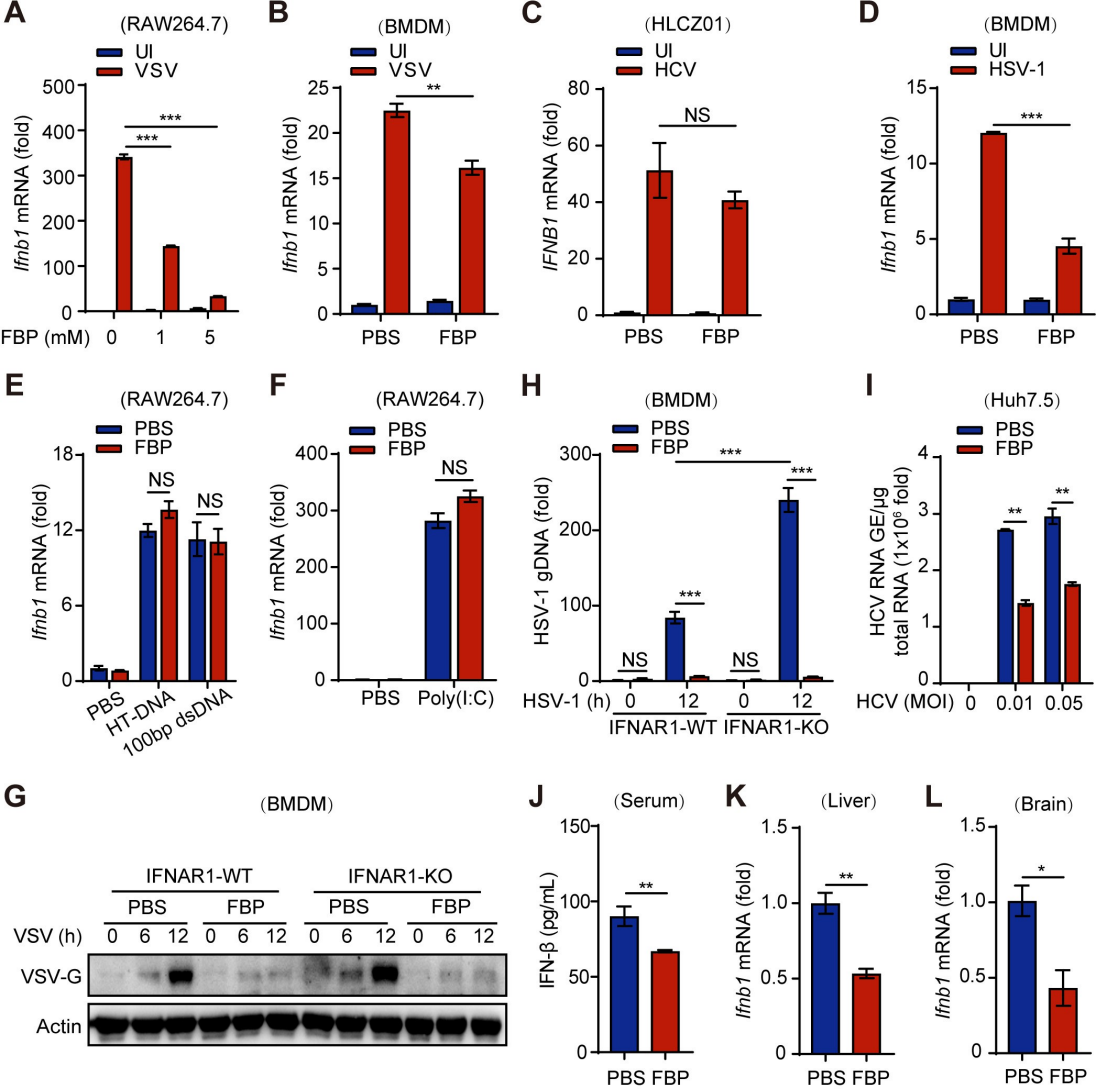
FBP suppresses viral infection largely independent of the type I IFN signaling pathway. (A and B) RAW264.7 cells (A) and BMDMs (B) were treated with the indicated concentration of FBP for 12 h and then infected with VSV (MOI, 0.1) for 10 h. The cells were collected, and the expression of *Ifnb1* mRNA was assessed by qPCR. (C) HLCZ01 cells were infected with HCV (MOI, 0.01) for 72 h and treated with 1 mM FBP for 12 h. The cells were collected, and the mRNA levels of *IFNB1* were assessed by qPCR. (D) BMDMs were treated with 1 mM FBP for 12 h and then infected with HSV-1 (MOI, 0.1) for 10 h. The cells were collected, and the mRNA levels of *Ifnb1* were assessed by qPCR. (E and F) RAW264.7 cells were treated with 1 mM FBP for 12 h and transfected with HT-DNA or 100 bp dsDNA for 8 h (E) or poly(I:C) (20 μg/mL) for 12 h (F), and the mRNA levels of *Ifnb1* were assessed by qPCR. (G and H) BMDMs from IFNAR1-WT and IFNAR1-KO mice were treated with 5 mM FBP for 12 h and then infected with VSV (MOI, 0.1) (G) or HSV-1 (MOI, 0.1) (H) for the indicated times. Western blot analysis of the VSV-G protein and qPCR analysis of HSV-1 gDNA levels were performed. (I) Huh7.5 cells were infected with HCV at the indicated MOI for 72 h and treated with 5 mM FBP for 12 h. The cells were collected, and the RNA levels of HCV were assessed by qPCR. (J-L) C57BL/6 mice were pretreated with FBP (500 mg/kg) or PBS (every 12 h for two days) and then intraperitoneally injected with VSV (J and K) or HSV-1 (L) (2×10^8^ PFU per mouse). FBP or PBS was injected at the same frequency during viral infection. Mice were sacrificed at 24 h (J and K) or 48 h (L) after infection to analyze serum IFN-β concentrations by ELISA (J) or the mRNA levels of *Ifnb1* in the liver (K) and brain (L) by qPCR. Data are presented as the mean ± SEM. NS, not significant, *p < 0.05; **p < 0.01; ***p < 0.001. one-way ANOVA. In (H), statistical analyses were performed with one-way ANOVA. In (A-F), (I) and (J-L), statistical analyses were performed with two-tailed Student’s t test.

We next investigated whether FBP inhibits viral infection by enhancing IFN-I-mediated innate immunity. Our analysis revealed that FBP did not increase the production of IFN-β in response to VSV, HCV, or HSV across various cell lines (Figs 3A–3D and S2E–S2I). Furthermore, FBP diminished the expression of the interferon-stimulated gene *Ifit1* and impaired the interferon-related signaling pathways activated by VSV (S2J and S2K Fig). These findings indicate that FBP does not activate the IFN-mediated innate immune response to inhibit viral infection.

Importantly, FBP also did not affect the expression of IFN-β induced by double-stranded DNA molecules such as HT-DNA or 100 bp dsDNA, nor by the double-stranded RNA analog poly(I:C), which lack replicative ability but are still infectious (Fig 3E and 3F). This prompted us to speculate that the alteration in virus-induced IFN production mediated by FBP may be a secondary consequence of its antiviral effects.

To further explore this, we assessed the effects of FBP on VSV and HSV infection in IFN-I receptor α subunit (IFNAR1)-knockout BMDMs, as well as HCV infection in Huh7.5 cells with disrupted IFN-I signaling (Fig 3G–3I). We found that FBP still suppressed viral replication in the presence of impaired IFN-I signaling (Fig 3G–3I). We also examined the time course of VSV-induced IFN production and observed that short-term FBP treatment enhanced IFN-β production, while long-term treatment had an inhibitory effect (S2L Fig). This pattern aligns with FBP’s role in promoting viral entry initially and inhibiting viral replication later, suggesting that FBP-induced changes in IFN levels are contingent on its regulatory role during the viral lifecycle.

*In vivo* experiments further demonstrated that FBP reduced serum and tissue IFN-β levels following VSV or HSV infection (Fig 3J–3L). Collectively, these results suggest that the inhibition of viral infection by FBP operates largely independently of the IFN-I signaling pathway.

### HMGB1 plays a crucial role in the FBP-mediated inhibition of viral replication

Our recent study demonstrated that FBP can inhibit tumor growth and enhance the sensitivity of tumors to chemotherapy by directly binding to HMGB1 [33]. HMGB1 has been implicated in regulating viral replication through various mechanisms, including direct interactions with viruses, modulation of autophagy, and functioning as DAMP [29, 35–39].

**Fig 4:**
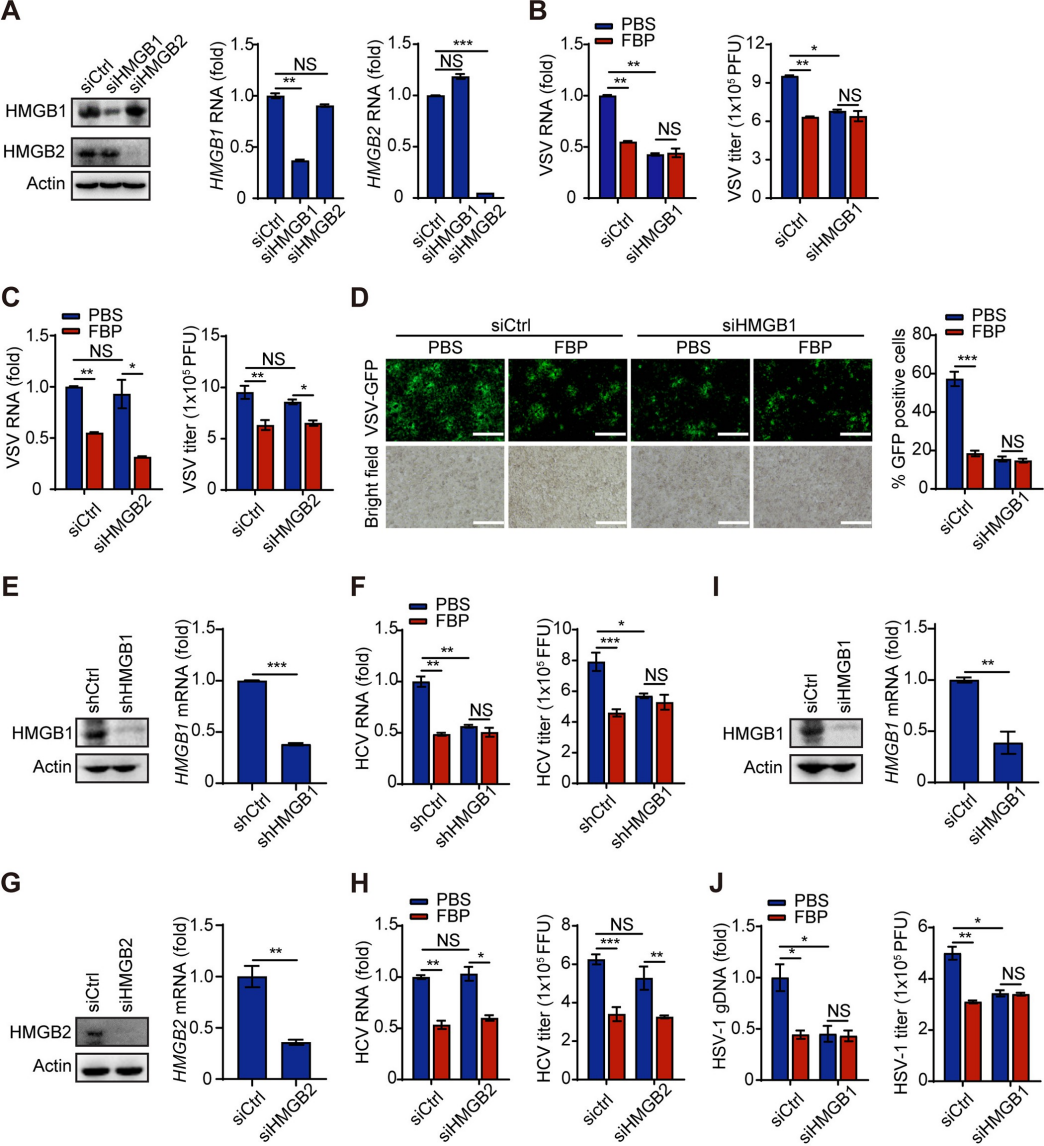
HMGB1 is essential for FBP-mediated inhibition of viral replication. (A-C) 293T cells were transfected with siControl (siCtrl), siHMGB1, and siHMGB2, followed by treatment with 1 mM FBP for 12 h and infection with VSV for 10 h. The expression of HMGB1 and HMGB2 was then analyzed using western blot and qPCR analysis (A). VSV RNA was assessed by qPCR analysis and VSV titers were assessed by plaque assay (B and C). (D) 293T cells were transfected with siControl and siHMGB1 and then treated with 5 mM FBP for 12 h, after which they were infected with VSV-GFP. The cells were then observed under a fluorescence microscope (left panel; scale bars, 100 μm; right panel, quantification of the percentage of GFP-positive cells). (E-H) Huh7.5 cells were transfected with shControl (shCtrl) and shHMGB1 (E and F) or siControl and siHMGB2 (G and H), infected with HCV (JFH-1 strain) (MOI, 0.01) for 72 h, and then treated with 1 mM FBP for 12 h. The cells were collected, and the expression of HMGB1 and HMGB2 was analyzed by western blot and qPCR analysis (E and G). HCV RNA was assessed by qPCR analysis and the HCV titers were determined as the average number of NS5A-positive foci (F and H). (I and J) 293T cells transfected with siCtrl or siHMGB1 were treated with 1 mM FBP for 12 h and then infected with HSV-1 (MOI, 0.1) for 10 h. Subsequently, the expression of HMGB1 was analyzed by western blot and qPCR analysis (I). HSV-1 gDNA was assessed by qPCR analysis and the HSV-1 titers were determined by plaque assay (J). Data are presented as the mean ± SEM. NS, not significant, *p < 0.05; **p < 0.01; ***p < 0.001. In (B), (C), (F), (H) and (J), statistical analyses were performed with one-way ANOVA. In right panels of (A), (E), (G) and (I), statistical analyses were performed with two-tailed Student’s t test.

Consequently, we investigated whether FBP could inhibit viral replication via HMGB1. We found that the inhibitory effect of FBP on VSV replication was reduced when both HMGB1 and HMGB2 were knocked down using a pan-siRNA (pan-siHMGB) (S3A and S3B Fig). In contrast, the inhibitory effect persisted when only HMGB2 was specifically knocked down in RAW264.7 cells (S3A and S3B Fig). Additionally, knocking down HMGB1—rather than HMGB2—abolished FBP’s ability to inhibit VSV replication, as evidenced by reduced levels of VSV RNA, decreased VSV titers measured by qPCR and plaque assays, and diminished fluorescence of VSV-GFP in 293T cells (Fig 4A–4D). Furthermore, the knockdown of HMGB1 also negated FBP’s inhibitory effects on HCV and HSV replication (Fig 4E–4J). These findings indicate that HMGB1, rather than HMGB2, plays a crucial role in mediating the inhibitory effects of FBP on viral replication.

### FBP primarily decreases the protein level of HMGB1 by promoting the lysosomal degradation of HMGB1 upon viral infection

**Fig 5:**
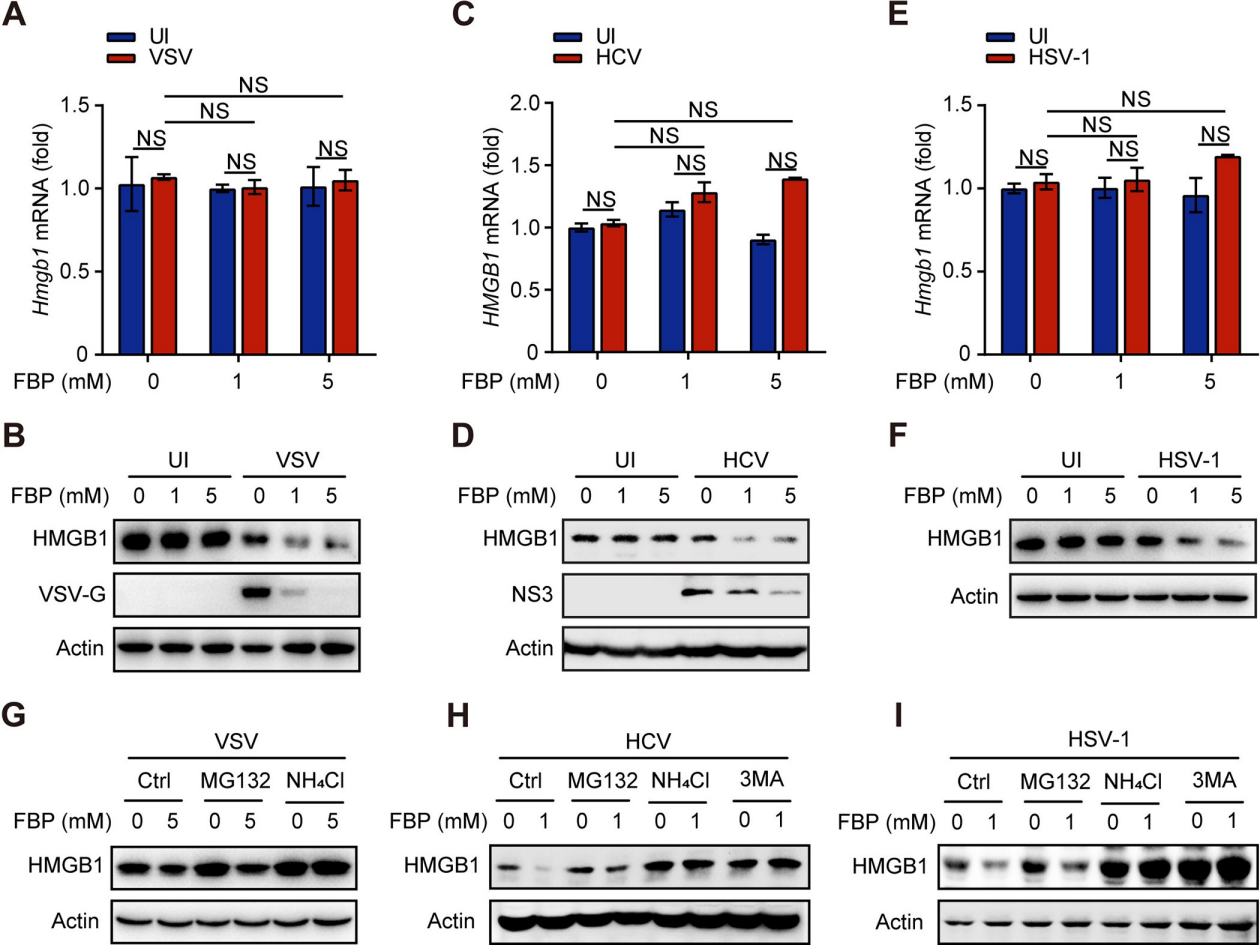
FBP primarily decreases the protein level of HMGB1 by promoting its lysosomal degradation upon viral infection. (A and B) RAW264.7 cells were treated with the indicated concentration of FBP for 12 h and infected with VSV (MOI, 0.1) for 10 h. The cells were collected, the mRNA levels of *Hmgb1* were assessed by qPCR (A), and HMGB1 and VSV-G expression was analyzed by western blot (B). UI, Uninfected. (C and D) Huh7.5 cells were infected with HCV (MOI, 0.01) for 72 h and treated with the indicated concentration of FBP for 12 h. The cells were collected, the mRNA levels of HMGB1 were assessed by qPCR (C), and HMGB1 and NS3 expression was analyzed by western blot (D). (E and F) RAW264.7 cells were treated with the indicated concentration of FBP for 12 h and infected with HSV-1 (MOI, 0.1) for 10 h. The cells were collected, and the mRNA levels of *Hmgb1* were assessed by qPCR (E). HMGB1 expression was analyzed by western blot (F). (G-I) 293T cells were treated with 5 mM FBP for 12 h and infected with VSV (MOI, 0.1) for 10 h (G). Huh7.5 cells were infected with HCV (JFH-1 strain) (MOI, 0.1) for 48 h and then treated with 5 mM FBP for 12 h (H). A549 cells were treated with 0 or 1 mM FBP for 12 h and infected with HSV-1 (MOI, 0.1) for 10 h (I). Then, the cells were treated with MG132 (25 μM), NH_4_Cl (10 mM) or 3-Methyladenine (3MA) (5 μM) for 6 h, after which HMGB1 expression was assessed by western blot. Data are presented as the mean ± SEM. NS, not significant. In (A), (C), and (E), statistical analyses were performed with one-way ANOVA.

To further explore how FBP inhibits viral replication through HMGB1, we investigated whether FBP regulates HMGB1 levels. Our results showed that exogenous FBP decreased the protein levels of HMGB1 without affecting its mRNA levels in multiple cell types infected with VSV, HSV, or HCV (Figs 5A–5F and S4A and S4B). Moreover, the reduction in HMGB1 protein levels induced by FBP was not reversed by pretreatment with the protein synthesis inhibitor cycloheximide (CHX) (Fig S4C–S4E), suggesting that HMGB1 is likely regulated at the posttranslational level.

HMGB1 can be degraded through both proteasome-dependent and lysosome-dependent pathways [40–45]. We discovered that the proteasome inhibitor MG132 did not prevent the FBP-mediated degradation of HMGB1 in virus-infected cells. In contrast, lysosomal inhibitors NH_4_Cl and 3-Methyladenine (3MA) effectively restored HMGB1 expression (Fig 5G–5I). Together, these findings indicate that FBP primarily reduces HMGB1 protein levels by promoting its lysosomal degradation during viral infection.

### FBP inhibits the binding of HMGB1 to the viral genome by interacting with HMGB1

HMGB1 consists of two homologous DNA/RNA binding domains, known as the A box and B box, along with an acidic C-terminal domain. In our previous study, we demonstrated that FBP can directly bind to HMGB1, disrupting the interaction between the HMGB1 A box and its C-tail. This disruption inhibits HMGB1 oligomerization and reduces its affinity for DNA and DNA adducts [33]. Consequently, we investigated whether FBP inhibits viral replication by affecting HMGB1’s binding to viral genomes.

**Fig 6:**
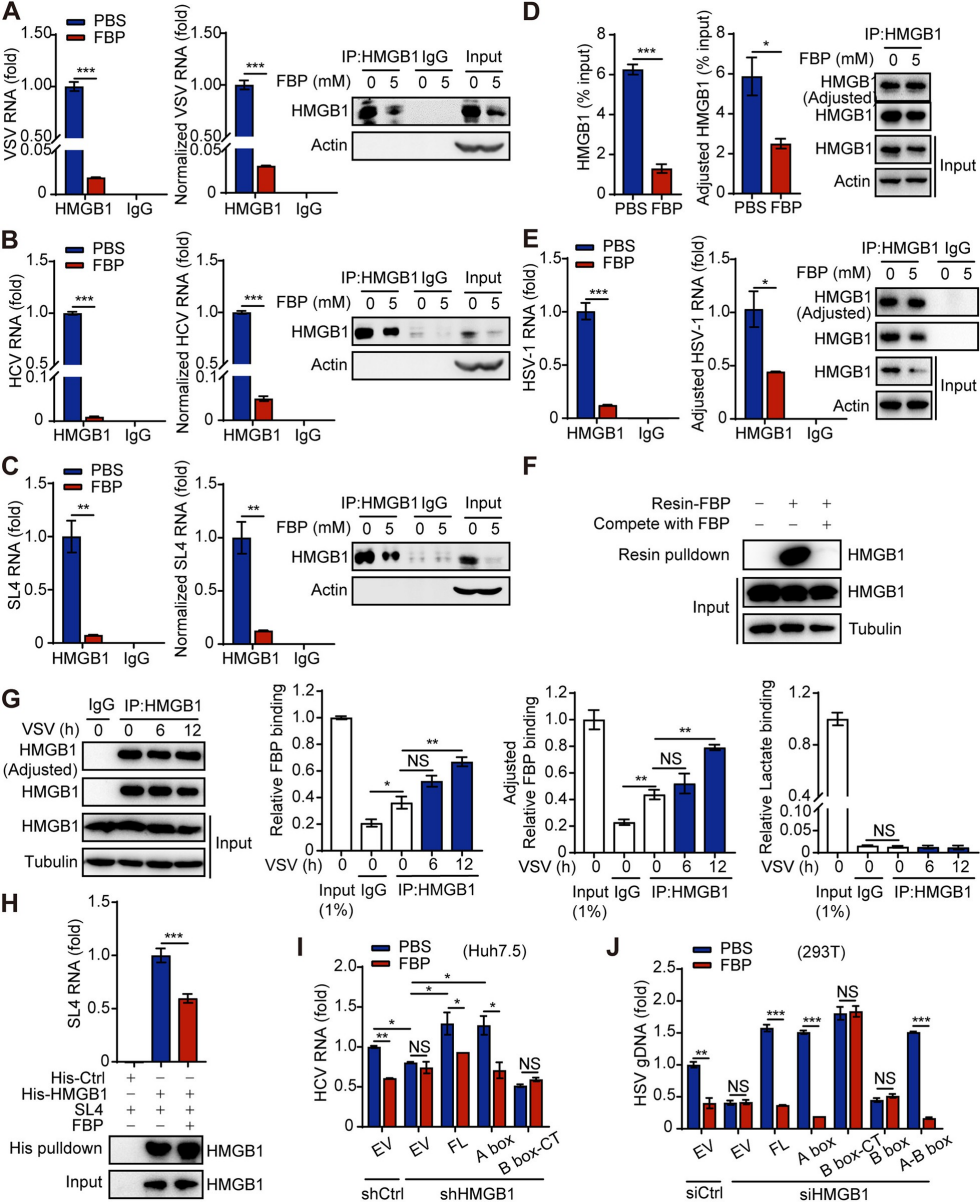
FBP inhibits the binding of HMGB1 to the viral genome by its interaction with HMGB1. (A-E) A549 cells were treated with FBP for 12 h, followed by infection with VSV (MOI, 0.1) for 6 h (A). Huh7.5 cells infected with HCV (JFH-1 strain) (MOI, 0.1) for 56 h were then treated with FBP for 12 h (B). Huh7.5 cells were transfected with an SL4-overexpression plasmid for 48 h and then treated with FBP for 12 h (C). 293T cells were treated with FBP for 12 h, followed by infection with HSV-1 (MOI, 0.1) for 10 h (D and E). Immunoprecipitation of HMGB1 with an anti-HMGB1 antibody and immunoblot analyses with the indicated antibodies were performed. RIP assays were then performed to assess the binding of VSV, HCV, SL4 or HSV-1 RNA to HMGB1 (A, B, C and E). ChIP assays were conducted to assess the binding of HSV-1 gDNA to HMGB1 (D). The levels of VSV, HCV, and SL4 RNA were normalized to the amount of immunoprecipitated HMGB1, as determined by densitometric analysis of western blots (referred to as ’normalized’; right panels of A, B, and C). The amount of immunoprecipitated HMGB1 was adjusted to comparable levels based on its expression in the input samples (referred to as ’adjusted’; right panels of D and E). (F) The cell lysate was incubated with resin-FBP, and the resin-bound proteins were eluted by free FBP and analyzed by immunoblotting with the indicated antibodies. (G) Colorimetric analysis of FBP or lactate binding in complexes was performed by immunoprecipitation (IP) using IgG or anti-HMGB1 antibody from virus-infected 293T cells, followed by immunoblotting analyses with the indicated antibodies. (H) The effect of FBP on the binding of purified HMGB1 protein to the HCV-SL4 RNA sequence was analyzed using His pulldown assay. Anti-HMGB1 antibodies were used for immunoblotting, and the level of the viral SL4 genome pulled down by HMGB1 was detected using qPCR. (I) Huh7.5 cells infected with HCV (JFH-1 strain) (MOI, 0.1) for 24 h were infected with lentivirus-shControl (shCtrl) or lentivirus-shHMGB1 for 24 h and then transfected with full-length or truncated HMGB1 for 36 h. The levels of HCV RNA were analyzed by qPCR. (J) 293T cells were transfected with siControl (siCtrl) or siHMGB1 for 24 h and then transfected with full-length or truncated HMGB1 for 36 h, followed by treatment with HSV-1 (MOI, 0.1) for 6 h. The gDNA levels of HSV-1 were assessed by qPCR. Data are presented as the mean ± SEM. NS, not significant, *p < 0.05; **p < 0.01; ***p < 0.001. In (I) and (J), statistical analyses were performed with one-way ANOVA. In (A-E), (G) and (H), statistical analyses were performed with two-tailed Student’s t test.

RNA binding protein immunoprecipitation (RIP) assays revealed that FBP inhibits the binding of HMGB1 to VSV and HCV RNA (Figs 6A and 6B and S5A). Our earlier study demonstrated that HMGB1 enhances HCV replication by interacting with the stem-loop SL4 in the untranslated region of the HCV 5’ UTR (35). Notably, we found that FBP significantly inhibits the binding of HMGB1 to HCV SL4 in Huh7.5 cells (Fig 6C). Importantly, the impact of FBP on inhibiting HMGB1’s binding to the viral genome was much greater than its effect on reducing HMGB1 protein levels. Even after normalizing the VSV and HCV RNA levels to the amount of immunoprecipitated HMGB1 determined by densitometry from western blots (referred to as ’normalized’), FBP still significantly inhibited the binding of HMGB1 to VSV and HCV RNA (Figs 6A–6C and S5A).

Based on our findings, we hypothesized that FBP may directly reduce the binding affinity of HMGB1 for viruses. To test this hypothesis, HMGB1 levels were adjusted to achieve comparable levels during immunoprecipitation based on input expression (referred to as ’adjusted’). Importantly, FBP still significantly inhibited HMGB1 binding to HSV gDNA, as demonstrated by a chromatin immunoprecipitation (ChIP) experiment combined with quantitative PCR (ChIP-qPCR) (Fig 6D). This suggests that the inhibition of HMGB1’s binding to HSV gDNA by FBP is not solely attributable to a reduction in HMGB1 protein levels, but also indicates a direct inhibitory effect of FBP on HMGB1’s interaction with HSV. Furthermore, we utilized RIP to investigate the effect of FBP on HMGB1’s binding to HSV RNA and found that FBP similarly inhibited this interaction (Fig 6E). These results suggest that FBP may play a role at two critical stages of the HSV lifecycle: transcription and replication.

To investigate whether FBP inhibits HMGB1’s binding to the viral genome through interaction with HMGB1, we covalently attached FBP to a primary amine-functionalized resin (referred to as ‘resin-FBP’) based on a previously established method to capture intracellular HMGB1 (33). The results indicated that resin-FBP could bind to HMGB1, and this binding was diminished by the presence of free FBP (Fig 6F). Colorimetric analysis of FBP and lactate binding in complexes immunoprecipitated by anti-HMGB1 antibody showed that FBP, rather than lactate, binds to HMGB1 in cells under physiological conditions (Fig 6G). Notably, VSV infection significantly enhanced this interaction at 6–12 h post-infection (Fig 6G). To further explore the effect of FBP on the binding of purified HMGB1 to the HCV genome, we synthesized the SL4 RNA sequence of HCV and performed a His pulldown assay. The result revealed that FBP reduced the binding of purified HMGB1 to the HCV SL4 genome (Fig 6H). Taken together, these findings indicate that FBP prevents HMGB1 from binding to the viral genome by interacting with HMGB1.

Moreover, the inhibitory effect of FBP on HCV and HSV replication was reversed by knocking down HMGB1 but could be restored by overexpressing full-length HMGB1 or the A box, but not the B box or C-terminal domain (Figs 6I and 6J and S5B–S5D). These findings indicate that the HMGB1 A box plays a critical role in the suppression of FBP-mediated virus replication.

## Discussion

In recent years, several studies have highlighted the protective role of FBP against various stresses, such as harmful chemicals, cold, ischemia-related diseases, and septic shock, both *in vitro* and *in vivo* [25, 34]. However, the involvement of FBP in viral infections has not yet been explored. Our current study demonstrates that the influence of FBP at different concentrations on VSV and HSV infections follows a bell-shaped curve, with no such effect observed on HCV. Notably, low doses of FBP (such as 10 μM) significantly increased the genome copy numbers of VSV and HSV, potentially by enhancing glycolysis or facilitating viral adhesion and entry, as supported by our experimental evidence. This effect may be attributed to FBP’s ability to disrupt membrane stability by reducing interactions among fatty acyl chains, leading to increased membrane permeability [46]. Conversely, high concentrations of FBP exceeding 1 mM consistently inhibit viral infections across multiple cell lines, suggesting that high concentrations of FBP may impact different stages of the viral life cycle, particularly viral replication, rather than just viral adhesion and entry.

When we knocked down or overexpressed FBP1 or PFKP to regulate the consumption and production of endogenous FBP, we observed that the concentrations of endogenous FBP influenced viral infection. Importantly, FBP’s inhibition of viral replication was largely independent of IFN-I signaling, unlike lactate, a downstream glycolytic product that suppresses RLR-mediated interferon production and enhances RNA viral infections by targeting MAVS [20]. This suggests that the antiviral effect of FBP is not mediated by lactate.

Knocking down HMGB1 completely abolished the ability of FBP to inhibit RNA/DNA viral infection, highlighting the critical role of HMGB1 in FBP’s antiviral action. Our findings indicate that FBP primarily promotes the lysosomal degradation of HMGB1, leading to reduced levels of HMGB1 protein during viral infection. HMGB1 can be degraded via both the lysosomal and proteasomal pathways, representing two distinct cellular mechanisms of protein degradation [40–45]. Numerous studies have indicated that HMGB1 is primarily located in the nucleus, where it is sequestered within an intracellular pool in unstimulated cells [47]. However, upon viral infection, HMGB1 is rapidly mobilized and translocated to the cytoplasm and extracellular environment [48, 49]. Additionally, FBP has been reported to interact with the AMPK-mediated lysosomal pathway [50, 51]. Our study demonstrated that the binding of FBP to HMGB1 is enhanced during viral infection (Fig 6G), which may facilitate lysosome-mediated degradation of HMGB1. Furthermore, viral infections can alter the pH and functionality of lysosomes [52–55], potentially impacting the stability and degradation of HMGB1. Further research is needed to elucidate the specific mechanism by which FBP facilitates the lysosomal degradation of HMGB1.

The results of this study demonstrate that FBP suppresses viral replication by inhibiting the binding of HMGB1 to the viral genomes, an effect that extends beyond a mere decrease in HMGB1 protein levels. Rescue experiments utilizing truncated domains of HMGB1 indicated that FBP may inhibit HCV and HSV replication by disrupting the interaction between the A box domain of HMGB1 and the viral genome. Previous reports have established that HMGB1 interacts with viral genomes, influencing viral replication [35, 36, 38, 39]. In alignment with these findings, our study showed that HMGB1 binds to the SL4 stem loop in the untranslated region of HCV via its A box domain, thereby promoting HCV replication.

Moreover, our earlier study demonstrated that FBP directly binds to HMGB1, disrupting the interaction between the HMGB1 A box and C-tail. This disruption inhibits HMGB1 oligomerization and reduces its affinity for DNA and DNA adducts (33). Notably, we also observed that the binding between FBP and HMGB1 exists in untreated cells and increases 6 to 12 h post-VSV infection. Additionally, FBP can competitively inhibit HMGB1’s binding to viral genomes.

HMGB1 may be implicated in the regulation of the viral replication cycle through various complex mechanisms. It has been reported that HMGB1 interacts with viral structural and functional proteins, influencing viral replication. For instance, the A box of HMGB1 can bind to the ribonucleoprotein of the influenza virus, enhancing the activity of the viral polymerase and promoting replication [37]. Additionally, the interaction between HMGB1 and the LANA protein of Kaposi’s sarcoma-associated herpesvirus (KSHV) is crucial for maintaining latent viral infection [56]. During herpesvirus infections, nuclear HMGB1 can bind to viral proteins, thereby facilitating herpesvirus replication. Furthermore, HMGB1 can be released into the extracellular environment, where it acts as a DAMP molecule, contributing to pro-inflammatory immune responses and disease progression in conditions such as systemic lupus erythematosus and rheumatoid arthritis [57–63]. Notably, the release of HMGB1 due to respiratory syncytial virus-induced necrotizing apoptosis has been identified as a critical factor in viral bronchiolitis [64]. Given these multifaceted roles, we cannot discount the possibility that HMGB1 may also participate in the suppression of FBP-mediated viral replication through additional complex mechanisms.

**Fig 7:**
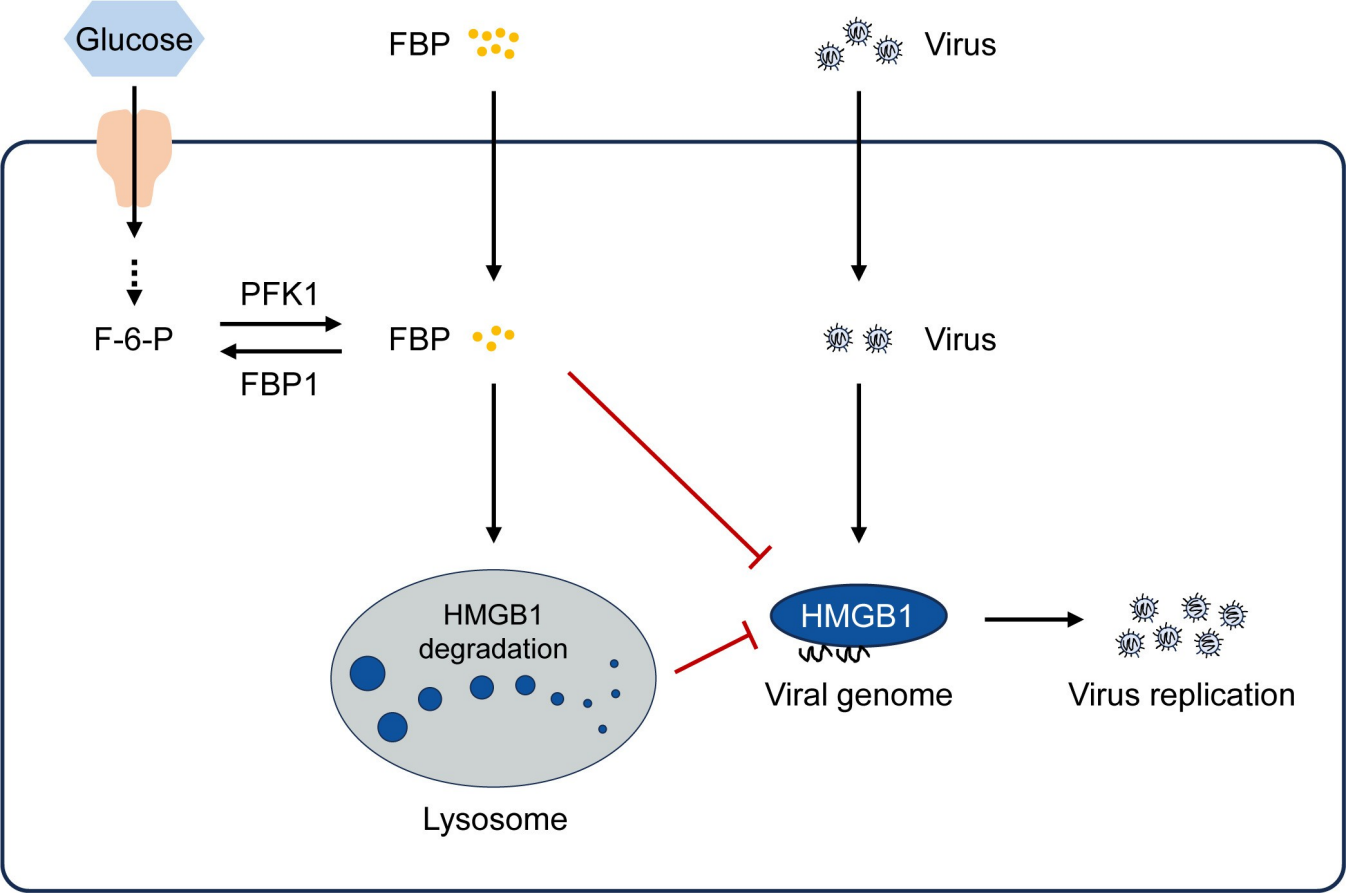
A schematic model illustrating how FBP regulates virus replication through HMGB1. FBP inhibits viral replication by promoting the lysosomal degradation of HMGB1 and interacting with it to prevent binding to the viral genome.

In summary, our findings uncover a novel function of the metabolic product FBP, which inhibits viral replication by promoting the lysosomal degradation of HMGB1 and interacting with it to prevent binding to the viral genome (Fig 7). This research highlights an important immunometabolism strategy that could be applicable to a range of human viral infectious diseases.

## Materials and methods

### Ethics statement

*Ifnar1*^-/-^ mice were generously provided by Dr. Chunsheng Dong (Soochow University, China). C57BL/6 mice were kept on a 12-hour light / 12-hour dark cycle at a controlled temperature of (22°C ± 2°C) and were given standard rat food and water in specific pathogen-free conditions. All animal experiments were approved by the Ethics Committee of Tianjin Medical University (Approval No. TMUaMEC 2018030).

### Antibodies and reagents

Primary antibodies against the following proteins were used for immunoblots: anti-HMGB1 (sc-56698, Santa Cruz Biotechnology), Anti-HCV-NS3 (ab13830, Abcam), Actin (A5541, Sigma), VSV-G(ab183497, Abcam), p-TBK1 (#5483, CST), TBK1 (#3504, CST), p-IRF3 (#4947, CST), IRF3 (#4302, CST), p-STAT1 (#10501-1-AP, ProteinTech), STAT1 (#14994S, CST), p-STAT2 (D155166-0025, Sangon Biotech), STAT2 (D261445-0025, Sangon Biotech), Tubulin (ab7792, Abcam), FBP1 (514824, Boster), PFKP (sc-514824, Santa Cruz Biotechnology), FLAG (#F1804, Sigma-Aldrich). Secondary antibodies were goat anti-mouse IgG (HRP-linked) (AP124P, Merck Millipore) and goat anti-Rabbit IgG (HRP-linked) (AP307P, Merck Millipore).

The following antibodies were used for immunofluorescence staining: the mouse monoclonal anti-HCV-NS5A antibody was a gift from Dr. Chen Liu (Yale University, United States); goat anti-mouse IgG (H + L) highly cross-adsorbed secondary antibody conjugated with Alexa Fluor 488 (R37120, Thermo Fisher Scientific).

### Cells, plasmids and viruses

E0771 and THP1 cells were cultured at 37°C under 5% CO_2_ in RPMI-1640 medium supplemented with 10% fetal bovine serum (FBS) and 1% penicillin-streptomycin. HEK293T and RAW264.7 cells were cultured at 37°C under 5% CO_2_ in Dulbecco’s modified Eagle’s medium (DMEM) supplemented with 10% fetal FBS and 1% penicillin-streptomycin. Bone marrow cells, isolated from the tibia and femur of mice, were cultured to induce differentiation into bone-marrow-derived macrophages (BMDMs) in DMEM with 10% FBS and 30% L929 supernatant at 37°C under 5% CO_2_ for 7 days. The HLCZ01 cell line, a hepatoma cell line supporting the entire life cycle of HCV and HBV, was previously established in our laboratory. Huh7.5 cells were kindly provided by Charles M. Rice (Rockefeller University, New York). Huh7.5-MASVR and HLCZ01 cells were cultured in collagen-coated tissue culture plates containing DMEM-F-12 medium supplemented with 10% FBS, 40 ng/ml dexamethasone (Sigma), insulin-transferrin-selenium (ITS) (Lonza), and penicillin–streptomycin (Thermo Fisher Scientific). Huh7.5 cells were propagated in DMEM supplemented with 10% FBS, 1% nonessential amino acid solution (Thermo Fisher Scientific), and 1% penicillin-streptomycin. Cell transfection with plasmids was conducted using Lipofectamine 293T (Beyotime), Via-Fect Transfection Reagent (Promega) or Lipofectamine 2000 (Invitrogen) in Opti-MEM medium (Thermo Fisher Scientific).

The FBP1 wildtype and mutant plasmids and PFKP plasmid were provided by Dr. Ting Wang (Tianjin Medical University, China). SL4 plasmid, full-length HMGB1 plasmid and HMGB1 domains, including the HMGB1 A box, B box, A-B box, and B box-CT were prepared based on our previous reference [36].

The HCV virus was generated using a plasmid encoding HCV JFH1 obtained from Takaji Wakita (National Institute of Infectious Diseases, Tokyo, Japan). In vitro transcription of the JFH1 genome was performed using a MEGAscript kit (Ambion, Austin, TX). The transcribed genome was then delivered into Huh7.5 cells via electroporation. Transfected cells were cultured in complete DMEM for the specified durations, with passage every 3 to 5 days. Supernatants were collected, filtered through a 0.45 μm pore-size filter device, and stored.

### RNA extraction and quantitative real-time PCR

Total RNA was isolated using TRIzol reagent (Invitrogen) and subsequently reverse-transcribed using a Reverse Transcription System (Bimake) prior to quantitative RT-PCR (qPCR) analysis. RT-PCR was conducted using the SYBR Green PCR Mix (Bimake) and suitable primers on an ABI 7300 Detection System. Expression levels were normalized to the control gene, *β-actin*, in each sample. For absolute quantitative analysis of HCV RNA, a standard curve was established using *in vitro* transcribed JFH1 RNA. The qPCR primers are listed in Table 1.

**Table 1:** Primers for real-time PCR.

Primer	Sequence
VSV-F	5’- ACGGCGTACTTCCAGATGG-3’
VSV-R	5’- CTCGGTTCAAGATCCAGGT-3’
HCV-F	5’- TCTGCGGAACCGGTGAGTA-3’
HCV-R	5’-TCAGGCAGTACCACAAGGC-3’
SL4-F	5’-GCGTTGGGTTGCGAAAGGCC-3’
SL4-R	5’-GGTGCACGGTCTACGAGACC-3’
HSV-1(EGFP)-F	5’-CAGTGCTTCAGCCGCTACCC-3’
HSV-1(EGFP)-R	5’-TGCCGTTCTTCTGCTTGTCG-3’
Mouse *Ifnb1*-F	5’-CCGAGCAGAGATCTTCAGGAA-3’
Mouse *Ifnb1*-R	5’-CCTGCAACCACCACTCATTCT-3’
Human *IFNB1*-F	5’-GCTTGGATTCCTACAAAGAAGCA-3’
Human *IFNB1*-R	5’-ATAGATGGTCAATGCGGCGTC-3’
Mouse *Ifit1*-F	5’-GTCCGGTTAAATCCAGAAGATCC-3’
Mouse *Ifit1*-R	5’-GCTTTGTCTACGCGATGTTTCC-3’
Human *HMGB1*-F	5’-TATGGCAAAAGCGGACAAGG-3’
Human *HMGB1*-R	5’-CTTCGCAACATCACCAATGGA-3’
Human *HMGB2*-F	5’-ATGGGTAAAGGAGACCCCAACAAGC-3’
Human *HMGB2*-R	5’-TTCTTCATCTTCATCCTCTTCCTCCTCATC-3’
Mouse *Hmgb1*-F	5’-GGCGAGCATCCTGGCTTATC-3’
Mouse *Hmgb1*-R	5’-GGCTGCTTGTCATCTGCTG-3’
Mouse *Hmgb2*-F	5’-GCTCGTTATGACAGGGAGATG-3’
Mouse *Hmgb2*-R	5’-TTGCCCTTGGCACGGTATG- 3’
*β-actin*-F	5’-CGTACCACTGGCATCGTGAT- 3’
*β-actin*-R	5’-AGGTAGTCAGTCAGGTCCCG-3’

### RNA interference

The target sequences of small interfering RNAs (siRNAs) were as follows: si*FBP1* (human), 5’-CGACCTGGTTATGAACATGTT-3’; si*PFKP* (human), 5’-CGAGAGGAGATTTCAAGATGC-3’; si*HMGB1* (human), 5’-GATGCAGCTTATACGAAATAA-3’; si*HMGB2* (human), 5’-GCTCAATACTAGCTTCAGTAT-3’; si*HMGB2* (mouse), 5’-GCTAAACTAAAGGAGAAGTAT-3’; pan-si*HMGB* (mouse), 5’-GTATGAGAAGGATATTGCT-3’. The transfection of siRNA was carried out using Lipofectamine RNAiMAX (Invitrogen) according to the manufacturer’s instructions. The efficiency of siRNA knockdown on the target protein was evaluated through qPCR and western blotting.

### Cell viability

Cell viability was assessed by CCK8 assay, in which cells were seeded in 96-well plates (2000 cells/well) or 24-well plates and different concentrations of FBP were treated for 0, 12 and 24 h. Then, 10 μl or 40 μl of Cell Counting Kit-8 (ApexBio Technology) was added to each well and incubated for 1–4 h at 37°C. The absorbance was measured at 450 nm through a microplate reader.

### Cell transfection

Cells were seeded in 10 cm or 12-well plates the day before transfection. Upon reaching 70% confluence, HEK293T cells were transfected using Lipofectamine 293T, while other cell types were transfected using Via-Fect Transfection Reagent or Lipofectamine 2000, following the manufacturer’s instructions. After 12 h, cells were replenished with fresh medium and maintained at 37°C in a 5% CO_2_ incubator.

### Lentivirus production and generation of knockdown cells

HEK293T cells plated in 10 cm dishes were transfected with 8 μg of packaging plasmid psPAX2, 2.7 μg of envelope plasmid pMD2G and 8 μg of target plasmid encoding shRNA using Lipofectamine 2000. Lentivirus supernatants were collected at 36 h, 48 h, 56 h and 72 h post-transfection and clarified by filtration through 0.45 μm syringe filters. Then the indicated cells were infected with the lentivirus. The target sequence of sh*HMGB1* was 5’-CCCAGATGCTTCAGTCAACTTCT-3’

### Western blotting

Cells were washed with PBS and lysed with RIPA buffer (Thermo Fisher Scientific) supplemented with protease inhibitor cocktail (Thermo Fisher Scientific). The lysates were incubated on ice for 30 min and centrifuged at 16000 g for 15 min at 4°C. Proteins were resolved on SDS–PAGE gels and transferred to polyvinylidene difluoride (PVDF) membranes (Merck Millipore). The PVDF membranes were then blocked with 5% skim milk and sequentially incubated with primary and secondary antibodies. The bound antibodies were detected using SuperSignal West Pico chemiluminescent substrate (Pierce, Rockford, IL).

### ELISA

The concentrations of IFN-β in the serum were measured using a mouse IFN-β ELISA kit (R&D Systems), according to the manufacturer’s instructions.

### Immunofluorescence

Cells were seeded into a confocal dish and fixed with 4% paraformaldehyde for 15 min at room temperature. The cells were washed with PBS, permeabilized for 15 min with 0.2% Triton X-100 in PBS for 10 min, blocked with goat serum (Thermo Fisher Scientific) for 30 min at room temperature, and then sequentially incubated with primary antibodies at 4°C for 12 h and fluorescence-labeled secondary antibodies (Invitrogen) (diluted in PBS to 1:500) at room temperature for 2 h. Nuclei were counterstained with DAPI (Vector Laboratories, Burlingame) for 5 min. Images were captured using a confocal microscope (Nikon). The HCV viral titers are presented as focus-forming units (FFUs) per milliliter, determined as the average number of NS5A-positive foci detected in Huh7.5 cells.

### Viral infection *in vivo*

In the study of viral infection *in vivo*, 6-8-week-old C57BL/6 mice were pretreated with FBP (500 mg/kg) or PBS (every 12 h for two days), and then intraperitoneally injected with VSV or HSV-1 virus (2×10^8^ PFU per mouse), respectively. FBP or PBS was injected at the same frequency during the viral infection. The mice were killed 24 h after VSV infection and 48 h after HSV-1 infection. The livers and brains of each mouse were taken for RNA or gDNA and virus titer analysis. In order to measure the titer of VSV in liver and the titer of HSV-1 in brain, the rapidly frozen tissue was weighed and homogenized in DMEM culture medium Then the suspension was centrifuged and the supernatant was used for TCID50 assay. The orbital sinus blood was taken for enzyme-linked immunosorbent assay (ELISA). In the survival curve, FBP treatment was continued until the first mouse died, and the survival of the mice was observed and recorded every day.

### TCID50 assay

Briefly, supernatants from tissue lysates or cultured cells were harvested and then used to infect Vero cell monolayers in 96-well plates with a series of ten-fold-diluted samples. After infection for 3 days, cells were stained for 30 min with 0.1% crystal violet. The number of wells displaying cytopathic effect (CPE) is determined, and the viral titers are calculated with the Spearman-Karber method formula [65].

### Lung histology

Lungs from control and virus-infected female mice were fixed with paraformaldehyde, embedded into paraffin, and then sectioned. Lung sections were stained with hematoxylin and eosin (H&E), dehydrated, and mounted. Images were obtained by light microscopy to detect histological change. We scored five histological features of the mouse lungs based on the McGuigan scoring system [66], including the alveolar neutrophils, interstitial neutrophils, hyaline membranes, proteinaceous debris, and alveolar septal thickening, to quantitatively assess the extent of virus-induced lung injury.

### RNA binding protein immunoprecipitation (RIP) assay

The cells were trypsinized to detach them, and the supernatant was discarded. The cells were washed by gently resuspending them in 1 ml PBS and pelleted by centrifugation at 3,000 g for 1 min. Then, the cells were resuspended in 1 mL 1% formaldehyde (diluted in PBS) for 10 min at room temperature. The cells were pelleted by centrifugation at 3,000 g for 1 min, and then resuspended in 1 mL 250 mM glycine solution (diluted in PBS) for 10 min. The cells were pelleted and washed with 500 μL PBS, and then the cells were lysed with RIPA buffer supplemented with a protease inhibitor cocktail and an RNase inhibitor on ice for 30 min. Immunoprecipitation was performed using antibodies against HMGB1 (sc-56698, Santa Cruz Biotechnology). Protein-RNA complexes binding to beads were eluted in PBS at 70°C for 45 min. The eluted material was lysed in ice-cold TRIzol reagent for qPCR.

### Chromatin immunoprecipitation (ChIP) assay

This experiment was performed according to the SimpleChip Plus Sonication Chromatin IP kit protocol (#56383, Cell Signaling). The cells were crosslinked with 1% formaldehyde for 10 min at room temperature, and stopped with 125 mM glycine. After sonicated treatment, the cell lysates were incubated with anti-HMGB1 antibody for immunoprecipitation. The sequences of HSV-1-ICP0 in both recovered DNA immunocomplexes and input DNA were detected by qPCR [67, 68]. The data is normalized to the corresponding DNA input control. The primer used was as follows: 5’-ATAAGTTAGCCCTGGCCCCGA-3’ (forward); 5’-GCTGCGTCTCGCTCCG-3’ (reverse).

### Resin pulldown assay

We followed the method described in a previous article to covalently link FBP to a primary amine-functionalized resin to capture intracellular HMGB1 [33]. First, HEK293T cell microspheres were washed once with PBS, and then resuspended in native lysis buffer (R0030, Solarbio), incubating at 4°C for 10 min. Next, the mixture was centrifuged at 4°C and 10,000g for 10 min. The cell extract was then incubated overnight at 4°C with either the FBP resin or a control resin. Afterward, the resin was washed four times with PBS and subjected to competitive elution with free FBP for 1 h. The bound proteins were eluted using a 1x loading buffer at 100°C for 10 minutes (heating the resin). Finally, immunoprecipitation was performed using a specific antibody.

### His pulldown assay

We incubated purified HMGB1 protein (10326-H08H, Sino Biological) and HCV-SL4 RNA sequence (Synbio technology) in buffer (100 mM Hepes, 50 mM NaCl, pH = 7) with PBS or FBP at 4°C for 2 h. Then, each sample was mixed with 10 μL TALON Metal Affinity Resins (635503, Takara) at 4°C for 1 h. After incubation, the resins were washed with PBS at 4°C three times. HMGB1 protein bands were detected by Western blot. RNA was extracted and the level of SL4 viral genomes pulled down by HMGB1 was detected by qPCR.

### Intracellular FBP or lactate measurement

The intracellular FBP or lactate concentrations were measured using a FBP ELISA kit (BC2245, Solarbio) or a lactate kit (E-BC-K044-M, Elabscience). The cells or IP complex was ultrasonically lysed and the supernatant was collected. The assays were performed in accordance with the reagent manufacturers’ protocols.

### Statistical analysis

All quantitative data are presented as the mean ± SEM of at least 2–3 independent experiments. The significance levels between the two groups were determined by two-tailed Student’s t-test. A one-way analysis of variance (ANOVA) was employed to compare data across multiple samples. Differences in survival rates were analyzed using the log-rank (Mantel-Cox) test. Statistical analysis was performed using GraphPad Prism 8.4 software. A *p* value <0.05 was considered statistically significant (NS, not significant (*p* ≥ 0.05); *p < 0.05; **p < 0.01; ***p < 0.001).

## Supporting information

S1 Fig FBP inhibits viral infection both *in vitro* and *in vivo*. (A) A549 cells were treated with the indicated concentration of FBP for 12 h and then infected with VSV (MOI, 0.1) for 10 h. The RNA levels of VSV were determined by qPCR and normalized to those in the FBP-untreated group. (B) RAW264.7 cells were treated with the indicated concentration of FBP for 12 h, and the level of intracellular FBP was measured by colorimetric assay. (C) E0771 cells were treated with the indicated concentration of FBP for 12 h and then infected with VSV (MOI, 0.1) for 10 h. The RNA levels of VSV were assessed by qPCR. (D) HLCZ01 cells were infected with HCV (JFH-1 strain) (MOI, 0.01) for 72 h and then treated with the indicated concentration of FBP for 12 h. The RNA levels of HCV were determined by qPCR and normalized to those in the FBP-untreated group. (E) Huh7.5-MAVSR cells were infected with HCV (MOI, 0.01) for 72 h and then treated with the indicated concentration of FBP for 12 h. The RNA levels of HCV were assessed by qPCR. (F-H) RAW264.7 cells (F), THP-1 cells (G) and E0771 cells (H) were treated with the indicated concentration of FBP for 12 h and then infected with HSV-1 (MOI, 0.1) for 10 h. The gDNA levels of HSV-1 were determined by qPCR, and the data were normalized to those of the FBP-untreated group. (I-O) Cell viability was assessed using CCK-8 assays after treatment with different concentrations of FBP for 0, 12 or 24 h. Data are presented as the mean ± SEM. NS, not significant, *p < 0.05; **p < 0.01; ***p < 0.001, two-tailed Student’s t test.
(DOCX)

S2 Fig FBP suppresses viral infection largely independent of the type I IFN signaling pathway. (A) RAW264.7 cells were treated with 5 mM FBP for 12 h and infected with VSV (MOI, 0.1) for the indicated times. The RNA levels of VSV were assessed by qPCR. (B-D) RAW264.7 cells pretreated with 5 mM FBP for 12 h were treated with VSV (MOI, 0.1) (B). HLCZ01 cells pretreated with 5 mM FBP for 12 h were inoculated with HCV (MOI, 0.1) (C). RAW264.7 cells pretreated with 5 mM FBP for 12 h were inoculated with HSV-1 (MOI, 0.1) (D). The cells were incubated at 4°C for 1 h, or at 37°C for 1 h, or at 4°C for 1 h and then at 37°C for 1 h. The levels of VSV RNA, HCV RNA or HSV-1 gDNA were analyzed by qPCR. (E-I) THP-1 cells and E0771 cells were treated with the indicated concentration of FBP for 12 h and infected with VSV (MOI, 0.1) (E and F) or HSV-1 (MOI, 0.1) (H and I) for 10 h. Huh7.5-MAVSR cells were infected with HCV (MOI, 0.01) for 72 h and treated with the indicated concentration of FBP for 12 h (G). The mRNA levels of *Ifnb1* were assessed by qPCR. (J and K) RAW264.7 cells were treated with the indicated concentration of FBP for 12 h and infected with VSV (MOI, 0.1) for 10 h (J) or the indicated times (K), followed by qPCR analysis of *Ifit* (J) or immunoblot detection with the indicated antibodies (K). (L) RAW264.7 cells were treated with 5 mM FBP for 12 h, then infected with VSV (MOI, 0.1) for the indicated times, followed by qPCR analysis of *Ifnb1* mRNA levels. Data are presented as the mean ± SEM. NS, not significant, *p < 0.05; **p < 0.01; ***p < 0.001, two-tailed Student’s t test.
(DOCX)

S3 Fig FBP plays a crucial role in inhibiting viral infection. (A and B) RAW264.7 cells were transfected with siControl (siCtrl), pan-siHMGB or siHMGB2, treated with 5 mM FBP for 12 h and infected with VSV (MOI, 0.1) for 10 h. Then, the expression of HMGB1 and HMGB2 was analyzed by western blot and qPCR (A). The RNA levels of VSV were assessed by qPCR (B). Data are presented as the mean ± SEM. NS, not significant, *p < 0.05; **p < 0.01. In (B), statistical analyses were performed with one-way ANOVA. In right panels of (A), statistical analyses were performed with two-tailed Student’s t test.
(DOCX)

S4 Fig HMGB1 plays a crucial role in the inhibition of viral replication mediated by FBP. (A and B) HLCZ01 cells were infected with HCV (MOI, 0.01) for 72 h and then treated with the indicated concentration of FBP for 12 h. Subsequently, the mRNA levels of HMGB1 were assessed by qPCR (A), and the protein levels of HMGB1 were analyzed by western blot (B). (C-E) HEK293T cells or A549 cells were treated with FBP for 12 h and infected with VSV (MOI, 0.1) (C) or with HSV-1 (MOI, 0.1) (E) for 10 h. Huh7.5 cells were infected with HCV (MOI, 0.01) for 72 h and treated with FBP for 12 h (D). The cells were then treated with CHX (100 ng/μL) for 6 h, followed by western blot analysis of HMGB1. Data are presented as the mean ± SEM. NS, not significant. In (A), statistical analyses were performed with one-way ANOVA.
(DOCX)

S5 Fig FBP primarily decreases the protein level of HMGB1 by promoting its lysosomal degradation upon viral infection. (A) HLCZ01 cells infected with HCV (JFH-1 strain) (MOI, 0.1) for 56 h were treated with FBP for 12 h. Immunoprecipitation of HMGB1 with an anti-HMGB1 antibody and immunoblot analyses with the indicated antibodies were performed. A RIP assay was then performed to test the binding of HCV RNA to HMGB1 The levels of HCV RNA were normalized to the amount of immunoprecipitated HMGB1, as determined by densitometric analysis of western blots (right panels). (B) Huh7.5 cells infected with HCV (JFH-1 strain) (MOI, 0.1) for 24 h were infected with lentivirus-shControl (shCtrl) or lentivirus-shHMGB1 for 24 h and then transfected with full-length or truncated HMGB1 for 36 h. The levels of HMGB1 were analyzed by western blot with the indicated antibody. (C) 293T cells were transfected with siControl (siCtrl) or siHMGB1 for 24 h and then transfected with full-length or truncated HMGB1 for 36 h, followed by HSV-1 infection (MOI, 0.1) for 6 h. The levels of HMGB1 were analyzed by western blot with the indicated antibody. (D) HLCZ01 cells were infected with HCV (JFH-1 strain) at an MOI of 0.1 for 24 h. Subsequently, the cells were infected with lentivirus-shControl or lentivirus-shHMGB1 for 24 h, followed by transfection with full-length or truncated HMGB1 for 36 h. The expression of HMGB1 protein was then analyzed by western blot. The levels of HCV RNA were analyzed by qPCR. Data are presented as the mean ± SEM. NS, not significant, *p < 0.05; **p < 0.01; ***p < 0.001. (D), one-way ANOVA. (A), two-tailed Student’s t test.
(DOCX)
