# Supplementary material for: Fructose-1,6-diphosphate inhibits viral replication by promoting the lysosomal degradation of HMGB1 and blocking the binding of HMGB1 to the viral genome
Source: PLoS Pathog. 2024 Dec 18;20(12):e1012782. doi: 10.1371/journal.ppat.1012782 (PMC11654956; doi:10.1371/journal.ppat.1012782)
Supplement: S1 Fig — (A) A549 cells were treated with the indicated concentration of FBP for 12 h and then infected with VSV (MOI, 0.1) for 10 h. The RNA levels of VSV were determined by qPCR and normalized to those in the FBP-untreated group. (B) RAW264.7 cells were treated with the indicated concentration of FBP for 12 h, and the level of intracellular FBP was measured by colorimetric assay. (C) E0771 cells were treated with the indicated concentration of FBP for 12 h and then infected with VSV (MOI, 0.1) for 10 h. The RNA levels of VSV were assessed by qPCR. (D) HLCZ01 cells were infected with HCV (JFH-1 strain) (MOI, 0.01) for 72 h and then treated with the indicated concentration of FBP for 12 h. The RNA levels of HCV were determined by qPCR and normalized to those in the FBP-untreated group. (E) Huh7.5-MAVSR cells were infected with HCV (MOI, 0.01) for 72 h and then treated with the indicated concentration of FBP for 12 h. The RNA levels of HCV were assessed by qPCR. (F-H) RAW264.7 cells (F), THP-1 cells (G) and E0771 cells (H) were treated with the indicated concentration of FBP for 12 h and then infected with HSV-1 (MOI, 0.1) for 10 h. The gDNA levels of HSV-1 were determined by qPCR, and the data were normalized to those of the FBP-untreated group. (I-O) Cell viability was assessed using CCK-8 assays after treatment with different concentrations of FBP for 0, 12 or 24 h. Data are presented as the mean ± SEM. NS, not significant, *p < 0.05; **p < 0.01; ***p < 0.001, two-tailed Student’s t test. (DOCX) [file ppat.1012782.s001.docx]

**S2 Fig**


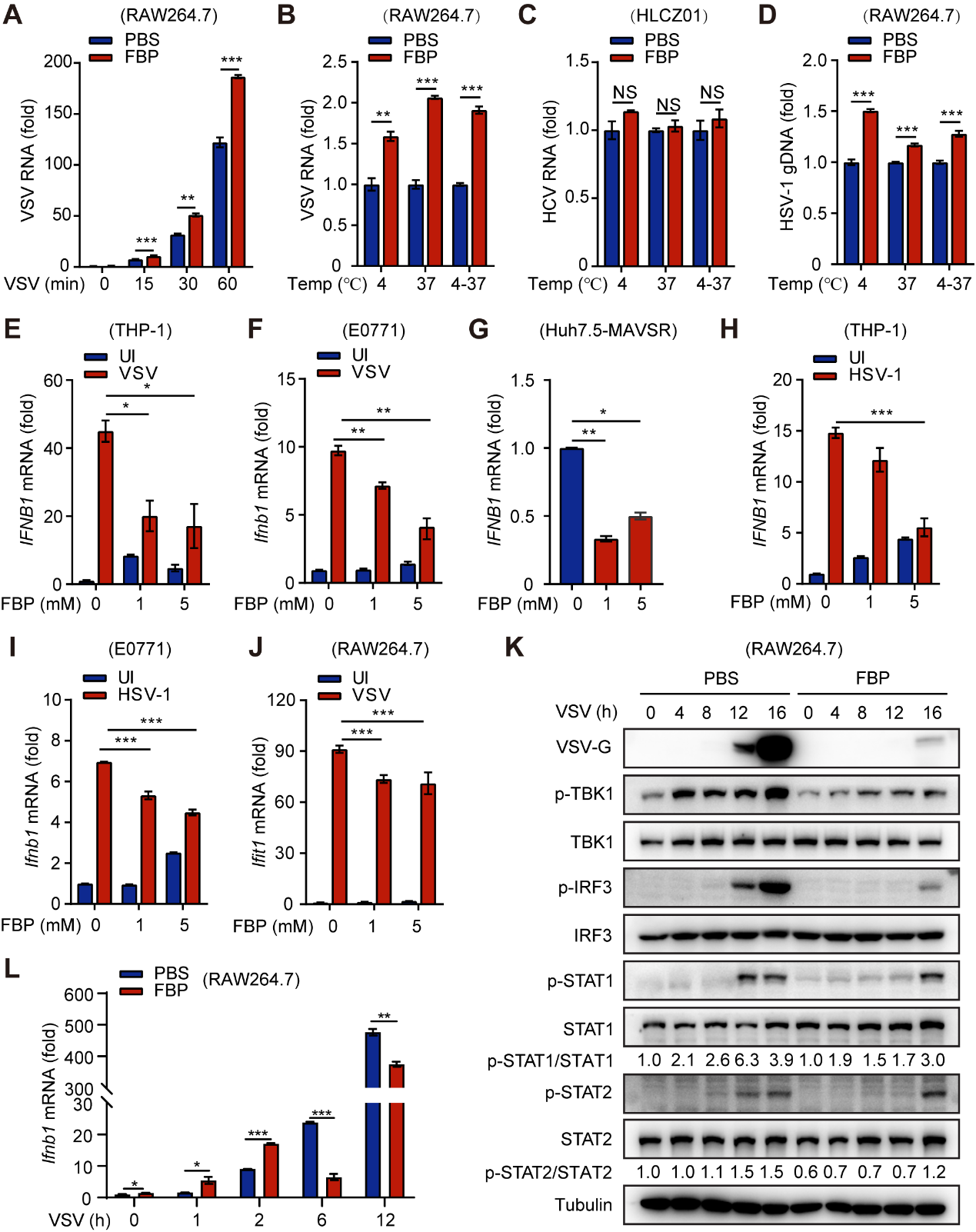


**S2 Fig. FBP suppresses viral infection largely independent of the type I IFN signaling pathway.**

(A) RAW264.7 cells were treated with 5 mM FBP for 12 h and infected with VSV (MOI, 0.1) for the indicated times. The RNA levels of VSV were assessed by qPCR.

(B-D) RAW264.7 cells pretreated with 5 mM FBP for 12 h were treated with VSV (MOI, 0.1) (B). HLCZ01 cells pretreated with 5 mM FBP for 12 h were inoculated with HCV (MOI, 0.1) (C). RAW264.7 cells pretreated with 5 mM FBP for 12 h were inoculated with HSV-1 (MOI, 0.1) (D). The cells were incubated at 4°C for 1 h, or at 37 °C for 1 h, or at 4 °C for 1 h and then at 37 °C for 1 h. The levels of VSV RNA, HCV RNA or HSV-1 gDNA were analyzed by qPCR.

(E-I) THP-1 cells and E0771 cells were treated with the indicated concentration of FBP for 12 h and infected with VSV (MOI, 0.1) (E and F) or HSV-1 (MOI, 0.1) (H and I) for 10 h. Huh7.5-MAVSR cells were infected with HCV (MOI, 0.01) for 72 h and treated with the indicated concentration of FBP for 12 h (G). The mRNA levels of *Ifnb1* were assessed by qPCR.

(J and K) RAW264.7 cells were treated with the indicated concentration of FBP for 12 h and infected with VSV (MOI, 0.1) for 10 h (J) or the indicated times (K), followed by qPCR analysis of *Ifit* (J) or immunoblot detection with the indicated antibodies (K).

(L) RAW264.7 cells were treated with 5 mM FBP for 12 h, then infected with VSV (MOI, 0.1) for the indicated times, followed by qPCR analysis of *Ifnb1* mRNA levels.

Data are presented as the mean ± SEM. NS, not significant, **p* < 0.05; ***p* < 0.01; ****p* < 0.001, two-tailed Student’s t test.
