# Supplementary material for: Fructose-1,6-diphosphate inhibits viral replication by promoting the lysosomal degradation of HMGB1 and blocking the binding of HMGB1 to the viral genome
Source: PLoS Pathog. 2024 Dec 18;20(12):e1012782. doi: 10.1371/journal.ppat.1012782 (PMC11654956; doi:10.1371/journal.ppat.1012782)
Supplement: S2 Fig — (A) RAW264.7 cells were treated with 5 mM FBP for 12 h and infected with VSV (MOI, 0.1) for the indicated times. The RNA levels of VSV were assessed by qPCR. (B-D) RAW264.7 cells pretreated with 5 mM FBP for 12 h were treated with VSV (MOI, 0.1) (B). HLCZ01 cells pretreated with 5 mM FBP for 12 h were inoculated with HCV (MOI, 0.1) (C). RAW264.7 cells pretreated with 5 mM FBP for 12 h were inoculated with HSV-1 (MOI, 0.1) (D). The cells were incubated at 4°C for 1 h, or at 37°C for 1 h, or at 4°C for 1 h and then at 37°C for 1 h. The levels of VSV RNA, HCV RNA or HSV-1 gDNA were analyzed by qPCR. (E-I) THP-1 cells and E0771 cells were treated with the indicated concentration of FBP for 12 h and infected with VSV (MOI, 0.1) (E and F) or HSV-1 (MOI, 0.1) (H and I) for 10 h. Huh7.5-MAVSR cells were infected with HCV (MOI, 0.01) for 72 h and treated with the indicated concentration of FBP for 12 h (G). The mRNA levels of Ifnb1 were assessed by qPCR. (J and K) RAW264.7 cells were treated with the indicated concentration of FBP for 12 h and infected with VSV (MOI, 0.1) for 10 h (J) or the indicated times (K), followed by qPCR analysis of Ifit (J) or immunoblot detection with the indicated antibodies (K). (L) RAW264.7 cells were treated with 5 mM FBP for 12 h, then infected with VSV (MOI, 0.1) for the indicated times, followed by qPCR analysis of Ifnb1 mRNA levels. Data are presented as the mean ± SEM. NS, not significant, *p < 0.05; **p < 0.01; ***p < 0.001, two-tailed Student’s t test. (DOCX) [file ppat.1012782.s002.docx]

**S3 Fig**


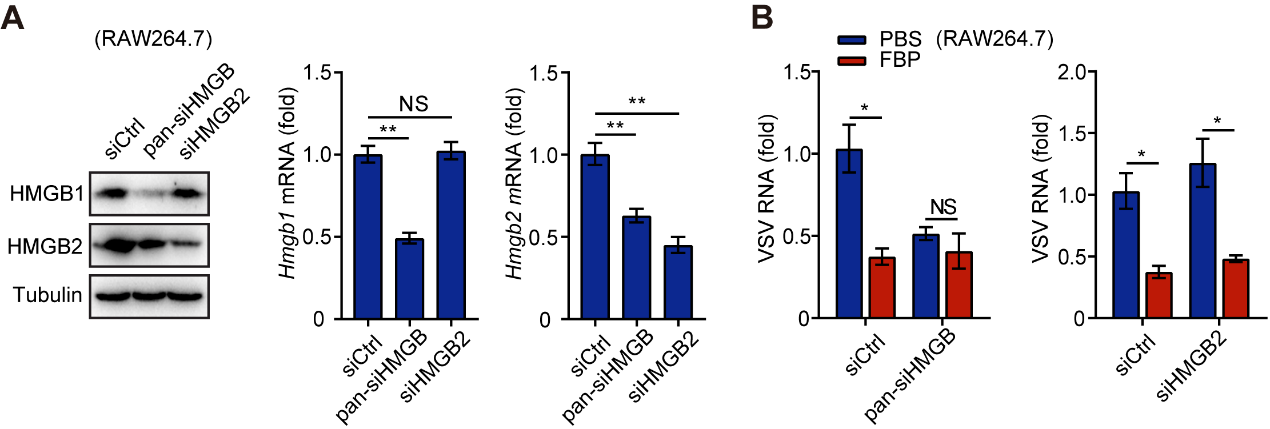


**S3 Fig. FBP plays a crucial role in inhibiting viral infection.**

(A and B) RAW264.7 cells were transfected with siControl (siCtrl), pan-siHMGB or siHMGB2, treated with 5 mM FBP for 12 h and infected with VSV (MOI, 0.1) for 10 h. Then, the expression of HMGB1 and HMGB2 was analyzed by western blot and qPCR (A). The RNA levels of VSV were assessed by qPCR (B).

Data are presented as the mean ± SEM. NS, not significant, **p* < 0.05; ***p* < 0.01. In (B), statistical analyses were performed with one-way ANOVA. In right panels of (A), statistical analyses were performed with two-tailed Student’s t test.
