# Supplementary material for: Fructose-1,6-diphosphate inhibits viral replication by promoting the lysosomal degradation of HMGB1 and blocking the binding of HMGB1 to the viral genome
Source: PLoS Pathog. 2024 Dec 18;20(12):e1012782. doi: 10.1371/journal.ppat.1012782 (PMC11654956; doi:10.1371/journal.ppat.1012782)
Supplement: S3 Fig — (A and B) RAW264.7 cells were transfected with siControl (siCtrl), pan-siHMGB or siHMGB2, treated with 5 mM FBP for 12 h and infected with VSV (MOI, 0.1) for 10 h. Then, the expression of HMGB1 and HMGB2 was analyzed by western blot and qPCR (A). The RNA levels of VSV were assessed by qPCR (B). Data are presented as the mean ± SEM. NS, not significant, *p < 0.05; **p < 0.01. In (B), statistical analyses were performed with one-way ANOVA. In right panels of (A), statistical analyses were performed with two-tailed Student’s t test. (DOCX) [file ppat.1012782.s003.docx]

**S4 Fig**


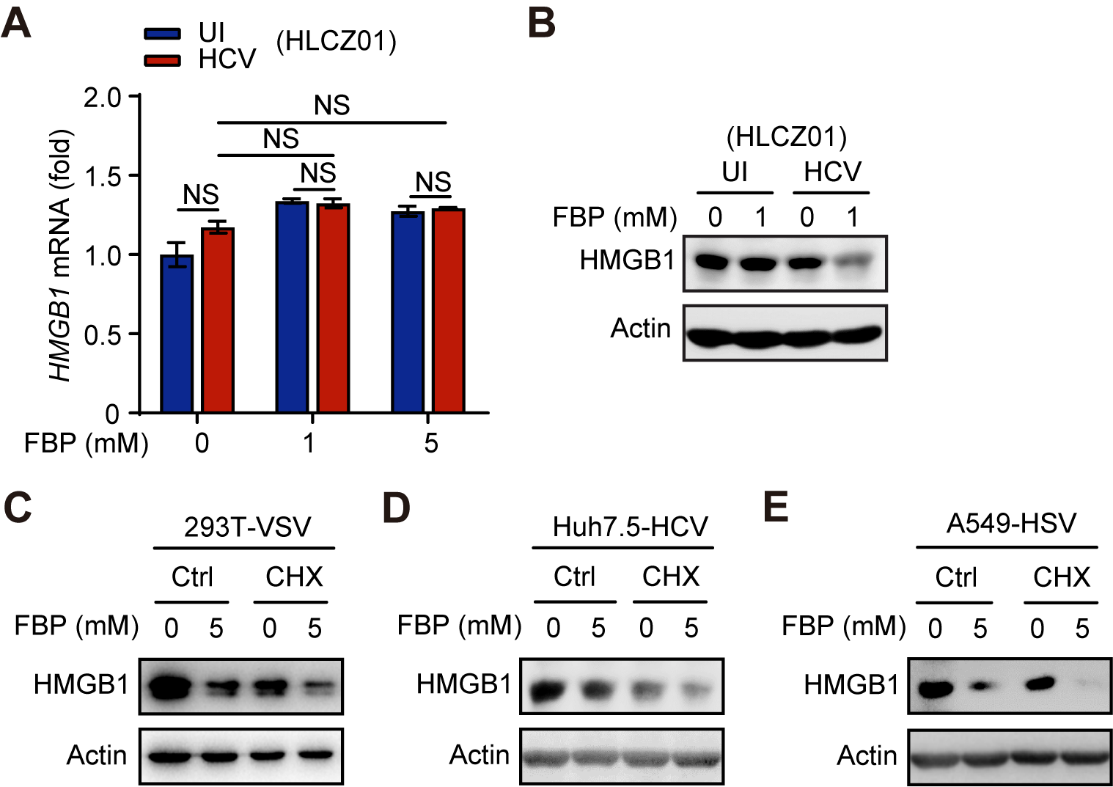


**S4 Fig. HMGB1 plays a crucial role in the inhibition of viral replication mediated by FBP**

(A and B) HLCZ01 cells were infected with HCV (MOI, 0.01) for 72 h and then treated with the indicated concentration of FBP for 12 h. Subsequently, the mRNA levels of HMGB1 were assessed by qPCR (A), and the protein levels of HMGB1 were analyzed by western blot (B).

(C-E) HEK293T cells or A549 cells were treated with FBP for 12 h and infected with VSV (MOI, 0.1) (C) or with HSV-1 (MOI, 0.1) (E) for 10 h. Huh7.5 cells were infected with HCV (MOI, 0.01) for 72 h and treated with FBP for 12 h (D). The cells were then treated with CHX (100 ng/μL) for 6 h, followed by western blot analysis of HMGB1.

Data are presented as the mean ± SEM. NS, not significant. In (A), statistical analyses were performed with one-way ANOVA.
