# Supplementary material for: Fructose-1,6-diphosphate inhibits viral replication by promoting the lysosomal degradation of HMGB1 and blocking the binding of HMGB1 to the viral genome
Source: PLoS Pathog. 2024 Dec 18;20(12):e1012782. doi: 10.1371/journal.ppat.1012782 (PMC11654956; doi:10.1371/journal.ppat.1012782)
Supplement: S4 Fig — (A and B) HLCZ01 cells were infected with HCV (MOI, 0.01) for 72 h and then treated with the indicated concentration of FBP for 12 h. Subsequently, the mRNA levels of HMGB1 were assessed by qPCR (A), and the protein levels of HMGB1 were analyzed by western blot (B). (C-E) HEK293T cells or A549 cells were treated with FBP for 12 h and infected with VSV (MOI, 0.1) (C) or with HSV-1 (MOI, 0.1) (E) for 10 h. Huh7.5 cells were infected with HCV (MOI, 0.01) for 72 h and treated with FBP for 12 h (D). The cells were then treated with CHX (100 ng/μL) for 6 h, followed by western blot analysis of HMGB1. Data are presented as the mean ± SEM. NS, not significant. In (A), statistical analyses were performed with one-way ANOVA. (DOCX) [file ppat.1012782.s004.docx]

**S5 Fig**


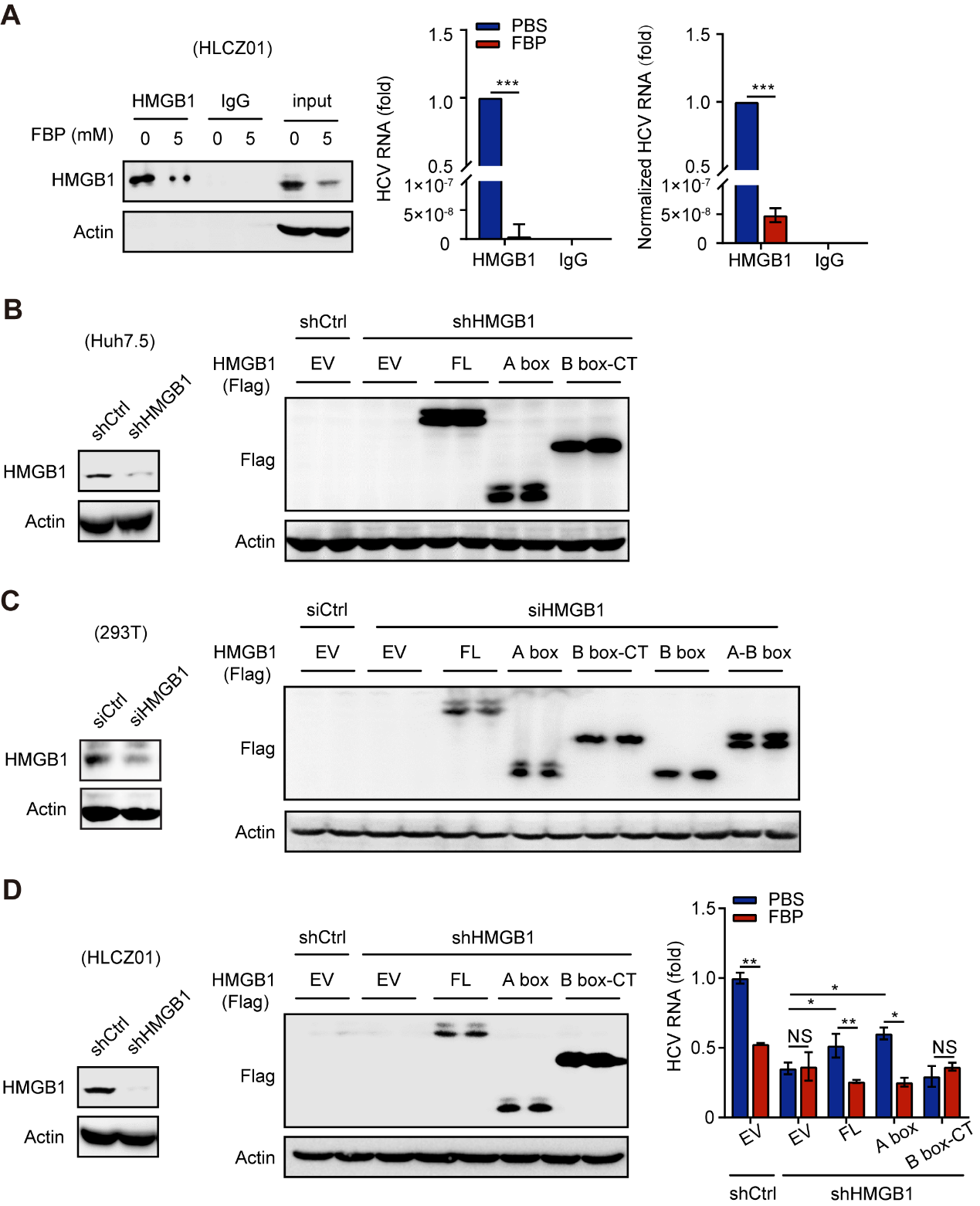


**S5 Fig. FBP primarily decreases the protein level of HMGB1 by promoting its lysosomal degradation upon viral infection.**

(A) HLCZ01 cells infected with HCV (JFH-1 strain) (MOI, 0.1) for 56 h were treated with FBP for 12 h. Immunoprecipitation of HMGB1 with an anti-HMGB1 antibody and immunoblot analyses with the indicated antibodies were performed. A RIP assay was then performed to test the binding of HCV RNA to HMGB1 The levels of HCV RNA were normalized to the amount of immunoprecipitated HMGB1, as determined by densitometric analysis of western blots (right panels).

(B) Huh7.5 cells infected with HCV (JFH-1 strain) (MOI, 0.1) for 24 h were infected with lentivirus-shControl (shCtrl) or lentivirus-shHMGB1 for 24 h and then transfected with full-length or truncated HMGB1 for 36 h. The levels of HMGB1 were analyzed by western blot with the indicated antibody.

(C) 293T cells were transfected with siControl (siCtrl) or siHMGB1 for 24 h and then transfected with full-length or truncated HMGB1 for 36 h, followed by HSV-1 infection (MOI, 0.1) for 6 h. The levels of HMGB1 were analyzed by western blot with the indicated antibody.

(D) HLCZ01 cells were infected with HCV (JFH-1 strain) at an MOI of 0.1 for 24 h. Subsequently, the cells were infected with lentivirus-shControl or lentivirus-shHMGB1 for 24 h, followed by transfection with full-length or truncated HMGB1 for 36 h. The expression of HMGB1 protein was then analyzed by western blot. The levels of HCV RNA were analyzed by qPCR.

Data are presented as the mean ± SEM. NS, not significant, **p* < 0.05; ***p* < 0.01; ****p* < 0.001. (D), one-way ANOVA. (A), two-tailed Student’s t test.
